# Dichloroacetate for Cancer Treatment: Some Facts and Many Doubts

**DOI:** 10.3390/ph17060744

**Published:** 2024-06-06

**Authors:** Tomas Koltai, Larry Fliegel

**Affiliations:** 1 Hospital del Centro Gallego de Buenos Aires, Buenos Aires 2199, Argentina; 2 Department of Biochemistry, University Alberta, Edmonton, AB T6G 2H7, Canada; lfliegel@ualberta.ca

**Keywords:** dichloroacetate, cancer, lactic acidosis, glioma, pyruvate dehydrogenase kinase, pyruvate dehydrogenase, pyruvate dehydrogenase phosphatase

## Abstract

Rarely has a chemical elicited as much controversy as dichloroacetate (DCA). DCA was initially considered a dangerous toxic industrial waste product, then a potential treatment for lactic acidosis. However, the main controversies started in 2008 when DCA was found to have anti-cancer effects on experimental animals. These publications showed contradictory results in vivo and in vitro such that a thorough consideration of this compound’s in cancer is merited. Despite 50 years of experimentation, DCA’s future in therapeutics is uncertain. Without adequate clinical trials and health authorities’ approval, DCA has been introduced in off-label cancer treatments in alternative medicine clinics in Canada, Germany, and other European countries. The lack of well-planned clinical trials and its use by people without medical training has discouraged consideration by the scientific community. There are few thorough clinical studies of DCA, and many publications are individual case reports. Case reports of DCA’s benefits against cancer have been increasing recently. Furthermore, it has been shown that DCA synergizes with conventional treatments and other repurposable drugs. Beyond the classic DCA target, pyruvate dehydrogenase kinase, new target molecules have also been recently discovered. These findings have renewed interest in DCA. This paper explores whether existing evidence justifies further research on DCA for cancer treatment and it explores the role DCA may play in it.

## 1. Introduction

### 1.1. Lactate and Cancer

Before understanding a putative role for DCA in cancer treatment, it is important to understand some basics about cancer and lactic acid metabolism.

Cancer cells differ from normal cells in many ways, including their metabolism, as described by Warburg [1,2]. Cancer metabolism is bound to the disease’s development and progression in such an intricate way that we may assume that there is no malignancy without the appropriate metabolic changes. Originally it was thought that the metabolic alterations mainly affected carbohydrate catabolic pathways, but now there is abundant and clear evidence that cancer’s metabolic alterations extend well beyond carbohydrates [3,4,5].

Among the many metabolic alterations in cancer, glycolytic metabolism, as opposed to oxidative metabolism, is one of the hallmarks of cancer [6]. Cancer “needs” a glycolytic phenotype to avoid cell death [7]. Apoptosis “needs” an oxidative phenotype [8]. Cells that depend primarily on the glycolytic pathway are therefore resistant to cell death [9,10,11], and cancer cells are particularly resistant to death.

The reversion of the glycolytic phenotype decreases proliferation and increases the apoptosis of malignant cells, as we shall further analyze in this review.

The main drivers of this metabolic transformation are the expression of oncogenic genes and hypoxia. Very early in a tumor’s development, these switch the preferential form of metabolism from oxidative to glycolytic metabolism. We describe the process as “preferential” because not all the cells in the tumor adopt glycolytic metabolism, some remain in oxidative metabolism. Furthermore, some non-malignant stromal cells are “enslaved” by the tumor and become glycolytic [12]. These produce lactic acid that is taken up and used by malignant cells that retain some oxidative properties as an energy source. It is important to note that even glycolytic cancer cells are not fully glycolytic and a certain percentage of glucose and lactate are metabolized through the mitochondrial oxidative pathway [13]. This is because glycolysis and oxidative metabolism are not mutually exclusive, but to a certain extent they are cooperative [14].

It is also important to understand lactic acid metabolism in cancer cells. Warburg found that the high production of lactic acid in cancer (60 times more than in normal cells, expressed in cubic millimeters per hour) is characteristic of cancer glycolytic metabolism [15,16]. The extrusion of this lactic acid from the cell is a vital activity for cell survival because if it were to remain inside, the intracellular pH would become very acidic and the cell would undergo acidic stress. Malignant cells need a slightly alkaline intracellular milieu for proliferation and biosynthesis [17,18]; therefore, it is essential for them to export this excess lactic acid. This is performed by specialized lactic acid transporters (MCTs). Of these, MCT1 and MCT4 are mainly responsible for lactate extrusion. Lactate transport is coupled with proton translocation, and so the export of lactate also implies an equimolecular number of exported protons. The extruded lactic acid is thus partly responsible for the extracellular acidity. As noted above, cancer cells can also import lactate, and MCT1 is the main lactate importer in malignant oxidative cells.

Cancer cells also have very important alterations in their intracellular and extracellular pH. With the generation of excess intracellular acid from lactate and other sources, there is simultaneously a very active extrusion of protons mediated by NHE1 (sodium-hydrogen exchanger-1) and other membrane ion channels [19]. These transporters prevent intracellular pH acidification. Additionally, there is a particular pH condition that is generated in cancer where the intracellular pH becomes slightly alkaline while the extracellular pH is quite acidic. This situation is exactly the opposite of what we find in normal tissues and is called the “inversion of the pH gradient” [20].

This inversion of the pH gradient is not exclusively a consequence of high lactic acid production and extrusion. Lactic acid is an important factor in the equation, and in normal blood, the lactate concentration does not go beyond 1 mM, while in the tumor microenvironment, it can rise to levels of 30–40 mM [21]. However, hypoxia and elevated proton extrusion are also strong mediators of extracellular acidity in the tumor microenvironment. In this regard, Newell [22] and Yamagata [23] have found that lactic acid is not essential for the creation of a highly acidic extracellular matrix (ECM) in tumors. 

Increased production and extrusion of lactic acid are the direct consequences of six phenomena:

(1) Malignant cells’ increased glucose uptake and metabolism;
(2) Glycolytic metabolism, which occurs even in the presence of oxygen (Warburg effect);
(3) Decreased activity of the pyruvate dehydrogenase (PDH) complex;
(4) Increased activity of pyruvate dehydrogenase kinases (PDKs);
(5) Increased expression and activity of the lactate extrusion proteins MCT1 and MCT4;
(6) Increased expression and activity of the glycolytic enzymes.

To summarize these causes, we can say that increased lactate is the result of increased glycolytic flux.

Not surprisingly, there have been studies of methods to block MCT transport as a form of chemotherapy [24,25,26,27,28]; however, there is no clinically approved drug for this purpose as yet. Lipophilic statins (atorvastatin and simvastatin) have shown MCT4 inhibitory effects at the experimental level, while the hydrophilic statins had negligible effects [29]. However, statins are not yet used clinically to inhibit MCTs.

Developing strong, clinically useful MCT inhibitors will not be easy, because normal cells need MCT activity. For example, muscle, retina, and other tissues use lactate as an energy source. This probably means that MCT inhibitors will show elevated toxicity towards normal cells. This is the case with AZD3965, an MCT1 inhibitor in an advanced state of research that has undergone phase I and II clinical trials [30].

Another more clinically useful approach could be to reduce lactic acid production, which would push the cell towards oxidative phosphorylation; this would be the supposed function of dichloroacetate (DCA).

The importance of lactic acid in cancer must be emphasized. It is true that many publications suggest that lactic acid is an end product or a “waste product” of glycolytic carbohydrate metabolism. However, lactate and lactic acid are neither end nor waste products.

Lactic acid serves as a fuel for oxidative malignant cells, as we have described above. The lactate shuttle that carries lactate from glycolytic cells to oxidative cells (referred to as lactophagic cells) means that lactate is not an end product of metabolism but can be further used by the appropriate cell types. Evidence that lactic acid is not a waste product with physiological or pathological roles, such as pro-tumoral effects, comes from the following studies showing that

(1) Lactate enhances the motility [31] and migration [32] of tumor cells.
(2) Lactate is a signaling molecule [33,34].
(3) It has immunosuppressive effects on tumors [35,36] by impeding dendritic cell activation [37,38] and impairing infiltrating CD8+ cytotoxic T-lymphocytes [39].
(4) Lactate induces pro-angiogenic effects [40,41,42,43,44].
(5) Lactate is strongly correlated with metastasis [45,46], and there is evidence that it can even promote metastasis [47].
(6) It can increase cancer cell stemness [48,49].
(7) Lactate activates HIF-1α in non-glycolytic cells (but not in glycolytic cells), which in turn further stimulates glycolysis and angiogenesis [50].
(8) Lactate has pro-inflammatory effects with increased NF-kB [51] and promotes IL17/IL23 pro-inflammatory pathways [52].
(9) Lactate has a positive correlation with radioresistance [53].
(10) It increases hyaluronan production involved in migration and growth [54].
(11) Lactate modulates the tumor microenvironment [55].
(12) Lactate plays a role in the transcription of oncogenes [56]. It has been shown that lysine lactylation of histone (the addition of a lactate residue to histone) introduces epigenetic modifications that stimulate gene transcription [57,58] (see below).
(13) Lactylation has also been found to be an important mechanism of post-translational modification of proteins associated with a poor prognosis of cancer progression [59].
(14) Lactate is an important source of energy for oxidative lactophagic cells [60].
(15) Pyruvate-to-lactate conversion by lactate dehydrogenase regenerates nicotinamide adenine dinucleotide (NAD+) in the cytoplasm [61], and NAD+ is an important metabolite for redox processes.

(For a review on lactate in cancer, see Kocianova et al. [62].)

All the above effects of lactate clearly show that it is not an innocent bystander, but an active tumor promoter. Some authors have called it an “oncogenic lacthormone” [63].

Therefore, the reduction of lactate production or its extrusion from cells becomes another important weapon in the fight against cancer. This is where DCA may have a contribution to make.

### 1.2. Dichloroacetate (DCA)

#### 1.2.1. Therapeutic History of Dichloroacetate 

DCA is an organic compound with the formula Cl_2_CHCO_2_H that is an analogue of acetic acid. DCA has been used for the treatment of lactic acidosis in acutely affected children and adults [64,65] and in congenital cases in children [66] since the 1970s [67]. The efficacy of DCA for treatment of lactic acidosis was examined in a randomized placebo-controlled study by Stacpoole et al. They did not find clear advantages of the treatment, although it decreased lactic acid concentrations in arterial blood by 20% or more and increased the arterial blood pH [68]. 

Another study examined children with lactic acidosis caused by malaria. Their treatment with DCA showed better results than in congenital cases [69]. A third study on DCA examined congenital lactic acidosis. This is a disease caused either by mutations of the pyruvate dehydrogenase (PDH) complex or by mutations in enzymes of the mitochondrial respiratory chain. Stacpoole et al. [70] examined over 200 pediatric and adult patients with congenital lactic acidosis, and DCA lowered the levels of lactic acid in blood. They fell at least 20% within 6 h of administration, although this did not improve clinical outcomes. DCA is still being investigated for the treatment of congenital lactic acidosis.

#### 1.2.2. DCA Enters the Oncology Terrain 

**Figure 1:**
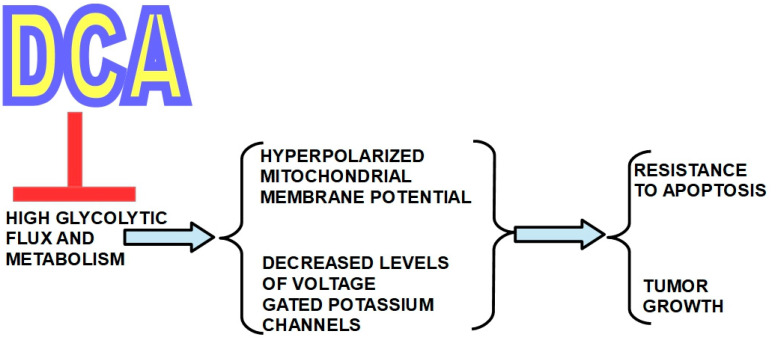
The first evidence of DCA’s antitumor effects and its mechanisms. Plas and Thompson have shown that glycolytic metabolism increased resistance to apoptosis [72].

In 2007, a group headed by Drs. Bonnet and Michelakis [71] called attention to DCA’s putative usefulness in cancer. They studied three highly glycolytic cell lines: glioblastoma (MO59K), lung adenocarcinoma (A549) and breast cancer cells (MCF7). The three cell types had a hyperpolarized mitochondrial membrane potential (ΔΨm) that was reversed to normal by an incubation with DCA in a dose-dependent manner. DCA also increased voltage-gated potassium channel (Kv1.5) expression, which was decreased in the malignant cells. The combination of a hyperpolarized ΔΨm and low expression of Kv1.5 is a consequence of high glycolytic flux and metabolism and induces resistance to apoptosis. They found that DCA induced apoptosis and decreased proliferation by restoring mitochondrial oxidative metabolism, without toxicity to normal cells (Figure 1).

**Figure 2:**
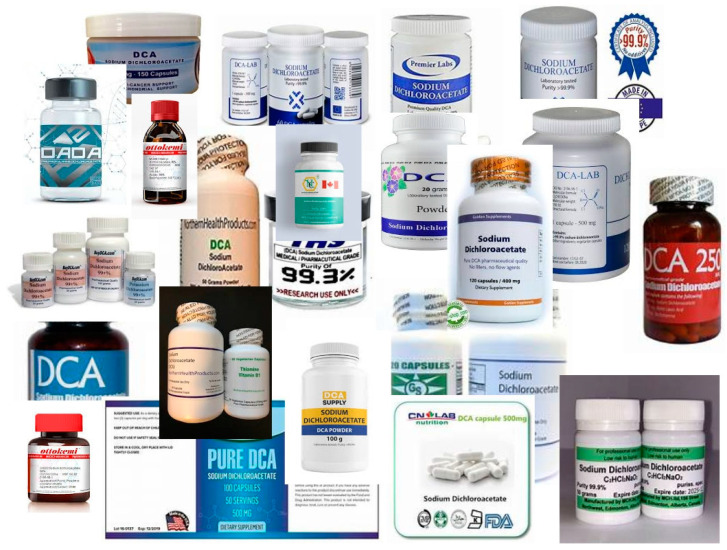
DCA is produced by many laboratories and is sold over the counter. The quality of the product from these laboratories is not well established. This explains many of the doubts about DCA’s real value as a therapeutic tool.

Since then, many clinics, usually called “DCA Clinics”, have opened, mainly in Canada and Germany. These clinics provide other naturopathic treatments for cancer, but the main therapy given is based on DCA. However, due to the lack of FDA approval, DCA has not entered mainstream oncological practice. Although DCA use was born as an alternative cancer treatment, unfortunately its real benefits in cancer are still unproven, although there is much evidence that supports the need for more testing. The fact that in many cases it has been co-opted by medically unqualified people raises doubts as to if it will ever be validated or accepted as an effective cancer treatment (Figure 2).

#### 1.2.3. Chemistry and Pharmacology of DCA

DCA is an investigational drug used to treat lactic acidosis, pulmonary hypertension, familial hyperlipidemia, and, more recently, cancer. Its use is unofficially accepted for the treatment of lactic acidosis in children with malaria in some African countries [73]. 

Humans are exposed to small amounts of DCA through drinking water. Chlorinated acetic acids, including DCA, are formed from organic material during water chlorination [74]. According to the US Environmental Protection Agency, DCA concentrations in drinking water range from 1 to 99 µg/L.

**Figure 3:**
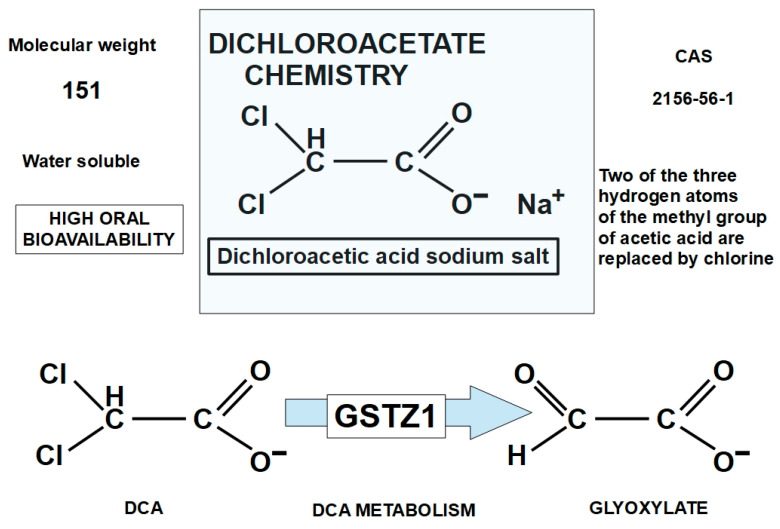
Chemical structure of DCA, also known as dichloroethanoic acid. It is a halogenated carboxylic acid and a structural analog of pyruvate. The lower panel shows its metabolization to glyoxylate by the enzyme glutathione transferase zeta 1 (GSTZ1), also known as maleylacetoacetate isomerase (MAAI). GSTZ1 dechlorinates DCA and GSTZ1 genetic variations influence the metabolic rate of DCA processing [75]. Glyoxylate is oxidized to oxalate, which is excreted in the urine. DCA has a high oral bioavailability [76].

The structure of DCA is shown in Figure 3. It is a small molecule and an organochlorine compound of acetic acid that is sometimes sold as a sodium salt. 

DCA is an orally available molecule that is quickly and almost completely absorbed by the digestive system [77]. It is initially distributed to the liver and muscles, and afterwards to other tissues [78]. DCA is metabolized by GSTZ1 (a glutathione transferase isoform) [79] (Figure 3). A total of 20% of DCA is bound to plasma proteins [80]. An example of how it is distributed throughout tissues was shown by James et al. In young adult rats given a single oral dose of 50 mg of sodium dichloroacetate per kg of body weight, the tissue distribution was muscle (11.9%), liver (6.19%), gastrointestinal tract (3.74%), fat (3.87%), and kidneys (0.53%), and the rest went to other tissues [81].

Effects on humans were examined after DCA was administered to 16 healthy individuals. With a 1 to 50 mg/kg IV infusion, concentrations of plasma glucose, lactate and alanine were examined. Plasma levels linearly followed those of the administered dose up to 30 mg/kg. A dose of 35 mg/kg was considered the most effective dose regarding lactic acid, which fell to 75% below baseline concentrations within 2 h of the infusion [82]. Alanine concentrations were also reduced, while the blood glucose concentration was not affected in these healthy individuals but it was reduced in diabetic patients through the stimulation of peripheral glucose utilization and inhibition of gluconeogenesis [83]. DCA also inhibited lipogenesis and cholesterol synthesis.

In diabetic patients with slightly elevated lactic acid levels, daily administration of oral DCA 50 mg/kg reduced alanine and lactic acid levels in plasma [84].

It should be noted that experimental administration of a single dose of DCA may yield misleading effects on the dose and metabolism compared with effects of repeated doses that could be used in patient treatment. Gonzalez-Leon et al. [85] have shown that DCA has a surprising feature when repeatedly administered to rats: it inhibits its own metabolism. This means that the chronic administration of DCA differs in its plasma concentration, toxicology and metabolism from the metabolism that occurs with single doses.

The half-life of DCA was examined in humans using IV infusion. With a 10 mg/kg infusion, the maximum plasma concentration achieved was between 19.9 μg/mL and 24.7 μg/mL, with a half-life of only 20 min. When the infused dose was increased to 20 mg/kg, the plasma concentration was between 57.3 and 74.9 μg/mL, with a half-life of 36 min. In dogs and rats, the half-life was much longer; the half-life of a 100 mg/kg dose was 4 h in rats and 24 h in dogs [86]. These marked differences among species call attention to the following:

(1) Doubts about the possibility of translating findings in other animals to humans;
(2) The importance of the inter-species differences in the clearance mechanism.

Confirming these inter-species differences, Maissenbacher et al. have shown that dogs have slower DCA metabolism and slower clearance than humans and rats due to greater inhibition of GSTZ1 [87].

The first dose is more rapidly cleared from plasma than subsequent doses, as Gonzalez-Leon et al. have shown [85]. This is probably due to GSTZ1 inhibition by DCA and is similar in all species. The decrease in DCA clearance by multiple doses is not a minor issue, because after successive doses, the initial clearance may be reduced to less than 25% of the initial one [88].

The metabolic pathway of DCA starts with dehalogenation to monochloroacetate and then glyoxylate. After dechlorination, degradation continues to glycine, and the final products are oxalate and carbon dioxide [89].

#### 1.2.4. Mechanism of Action

The best known and main mechanism of action of DCA seems to be the inhibition of pyruvate dehydrogenase kinase (PDK) and its isoforms [71,75,77,78,82,89]. This inhibition increases the flux of pyruvate into the mitochondria, promoting glucose oxidation instead of glycolysis [90].

Pyruvate dehydrogenase kinase (PDK) is a kinase that inactivates the pyruvate dehydrogenase enzyme (PDH) complex through phosphorylation. By downregulating the activity of this complex, PDK decreases the oxidation of pyruvate in mitochondria and increases the conversion of pyruvate to lactate in the cytosol. 

PDH is regulated by two systems:

(a) An inhibitory system represented by the four isoforms of PDK;
(b) A “disinhibitory” system represented by the two PDH phosphatases (see below).

Other mechanisms of action of DCA in cancer have been reported in the medical literature. Stockwin et al. [91] found that the cytotoxicity of DCA was only achieved in those cells that suffered mitochondrial DNA mutations that “condemn” them exclusively to the glycolytic pathway. (This would explain the synergy between DCA and metformin, see below.)

## 2. The PDH Complex

### 2.1. PDH Complex

**Figure 4:**
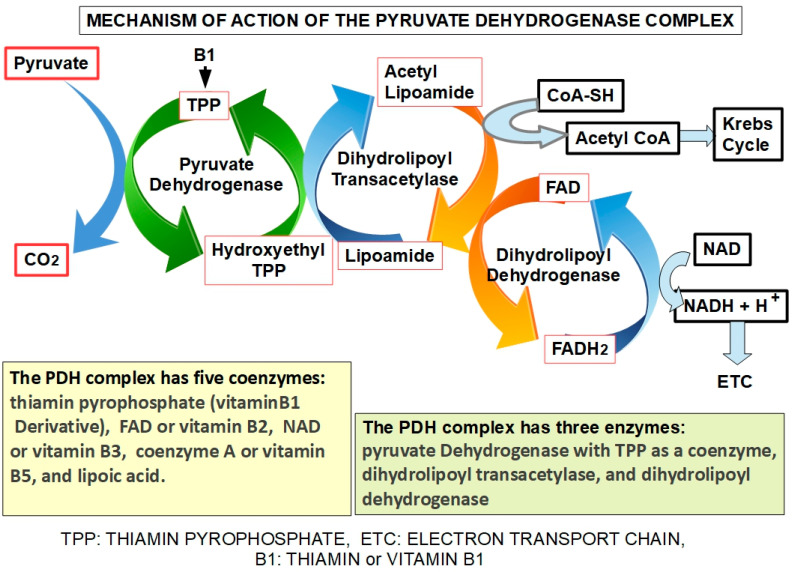
The PDH enzymatic complex introduces pyruvate into the Krebs cycle after removing a molecule of CO_2_ and binding it to coenzyme A, forming acetyl-CoA.

The PDH complex is the gate for pyruvate to enter the oxidative metabolic pathway. It is prudent to review the complex and its regulation. This enzymatic complex is formed by three enzymes. The first, pyruvate dehydrogenase, decarboxylates pyruvate, forming hydroxyethyl thiamin. Thiamin acts as a coenzyme (Figure 4). The second enzyme, dihydrolipoyl transacetylase, transfers the acetyl group to lipoamide and then to coenzyme A, forming acetyl-CoA that enters the Krebs cycle. The third enzyme, dihydrolipoyl dehydrogenase, captures two protons that are transferred to FAD (Figure 4). The PDH complex is regulated by phosphorylation by pyruvate dehydrogenase kinases and dephosphorylation by pyruvate dehydrogenase phosphatases. Insulin is a strong activator of the PDH complex [92,93].

### 2.2. PDK Family of Enzymes

The PDK enzymatic complex has been called the gatekeeper of mitochondria [94].

PDK was cloned and sequenced by Popov et al. in 1992 [95] and it functions as a histidine protein kinase. Four isoforms have been identified in humans: PDK1, PDK2, PDK3 and PDK4 [96,97]. PDK1 is under modulation by HIF-1α and cMyc [98,99] as part of the cellular adaptation to hypoxic conditions. PDK2, PDK3 and PDK4 seem to be regulated by the peroxisome proliferator activation receptors β/δ (PPAR β/δ) [100,101].

P53 negatively regulates PDK2 [102], thus promoting oxidative metabolism.

**Figure 5:**
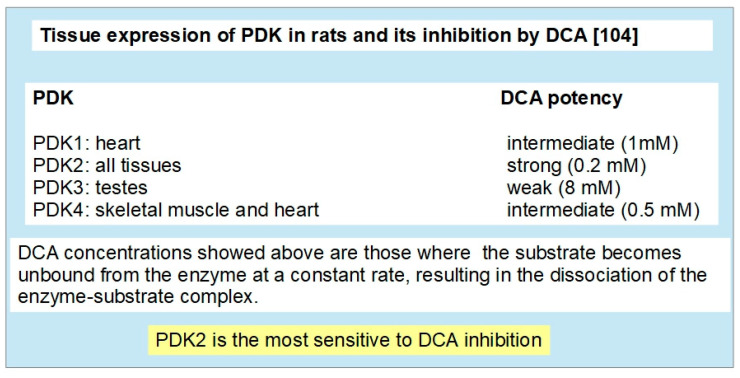
Differences in DCA potency according to the PDK isoform.

The mechanism by which PDKs inhibit PDH is serine phosphorylation at site 1 (there are three possible sites, but only site 1 is responsible for full deactivation of the PDH complex [103]). All the PDKs can phosphorylate PDH, but PDK2 has the greatest phosphorylation activity in vivo [104] (Figure 5). P53 is another negative regulator of PDK2 [105].

### 2.3. Inhibition of PDKs

PDK1 and PDK3 can be inhibited by different compounds. The mechanism of action of the investigational drug AZD7545 is through binding the lipoyl-binding pocket of the enzyme while DCA binds the interior of the helix bundle in the N-terminal domain, promoting conformational changes. Radicicol, another inhibitor, binds to the ATP-binding pocket [106]. 

**Figure 6:**
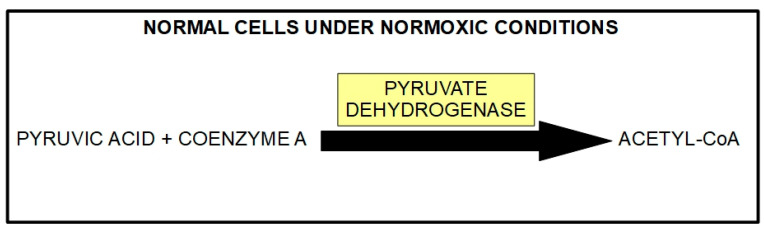
Pathway of pyruvic acid in normal cells during normoxia.

The downstream mechanism of DCA inhibition of PDK is to “force” the cancer cell to abandon its preferred metabolic process (aerobic glycolysis). This allows the PDH complex to drive pyruvic acid towards the mitochondrial tricarboxylic cycle (Figure 6). 

In cancer cells with elevated glycolytic metabolism, PDH is inhibited through phosphorylation by PDK. Thus, the chemical reaction shown above (Figure 6) does not take place, and pyruvate is transformed into lactic acid through the enzymatic activity of lactic dehydrogenase (LDH). This means that the pyruvate that normally goes into mitochondria as acetyl-coenzyme A and proceeds to the Krebs cycle does not do so in cancer cells. Instead, it remains in the cytosol and is converted into lactic acid. The lactic acid is then extruded from the cell through the activity of monocarboxylate transporters (MCTs).

**Figure 7:**
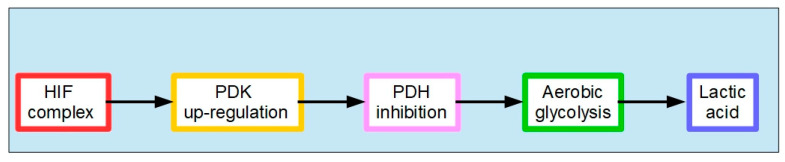
Carbohydrate metabolism in cancer cells. PDK is controlled by the HIF (hypoxia-inducible factor) complex. Upregulation of PDK results in PDH inhibition and elevated aerobic glycolysis and lactic acid levels (see also Section 2.2).

**Figure 8:**
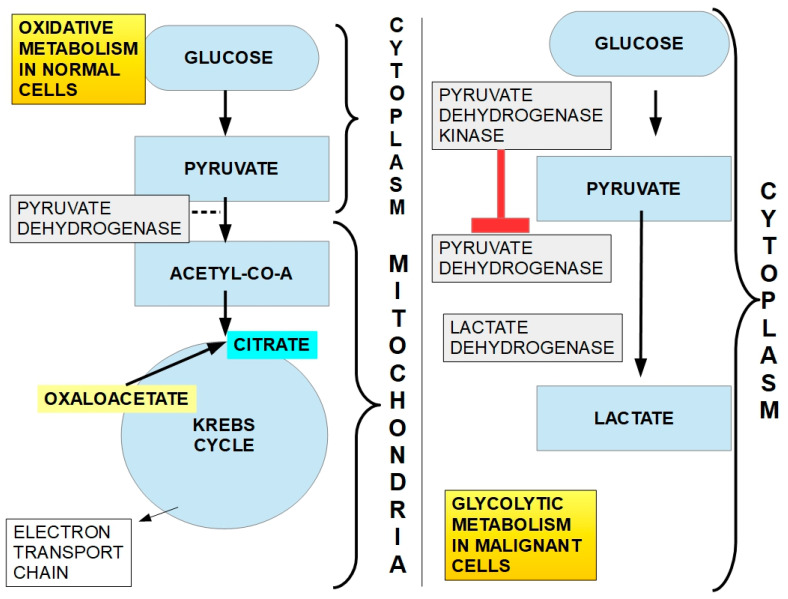
Glucose metabolism in normal cells (oxidative metabolism, left side) and in cancer cells (glycolytic metabolism, right side). In normal cells, the chemical end products are CO_2_ and H_2_O. In cancer cells, the end product is lactic acid, which is extruded from the cell through MCTs. The activity of PDH is the cornerstone that defines which way metabolism will go. In this case, mitochondrial PDH irreversibly decarboxylates pyruvate to acetyl-coenzyme A, linking glycolysis to the tricarboxylic acid cycle (left panel). This is a critical step in carbohydrate metabolism where a decision point is reached: the glycolytic or oxidative pathway. If PDH is inhibited by phosphorylation by PDK (right panel), pyruvate will be taken care of by the glycolytic pathway. An important fact is that malignant cells show 2-to-20-fold higher glucose intake, which means an average 60-fold increase in lactate production. When this lactate is extruded, it has an impact on extracellular acidity. However, lactate does not seem to be the main cause of extracellular acidity (Figure 9).

Normal cells can also use this same mechanism when oxygen levels are very low. In this case, it is called anaerobic glycolysis. When oxygen levels improve, the handling of pyruvic acid returns to the normal pathway (Figure 6). The interesting phenomenon is that cancer cells use this mechanism even when oxygen levels have improved. Almost 100 years ago, Crabtree [107] and Warburg, respectively, showed that it occurs in yeast and in mammalian cancer cells. This phenomenon is called aerobic glycolysis or the Warburg effect. Not only does this occur in cancer cells, but they are highly dependent on it. Figure 7 illustrates the pathway leading to production of lactic acid in cancer cells and Figure 8 shows the major metabolic pathways in normal versus malignant cells.

### 2.4. Pyruvate Dehydrogenase Phosphatases (PDPs)

PDH is not permanently inactivated by phosphorylation. The enzyme is reactivated via the actions of two PDPs (PDP1 and PDP2) that dephosphorylate PDH. The PDPs are regulated by magnesium and calcium [109].

### 2.5. Mechanism of Action of DCA

The main mechanism of the anticancer activity of DCA is the inhibition of mitochondrial pyruvate dehydrogenase kinase. This prevents the inhibition of pyruvate dehydrogenase and therefore facilitates the switch from glycolytic to oxidative metabolism. The inhibition by DCA is not competitive with ATP [110]. An important consequence of this conversion to oxidative metabolism by DCA is an increase in caspase-mediated apoptosis, preventing the increase in cell survival that glycolytic metabolism enhances [111]. DCA was first recognized as a PDK inhibitor by Whitehouse and Randle fifty years ago [112]. They wrote “*Evidence is given that dichloroacetate may facilitate the conversion of pyruvate dehydrogenase from an inactive (phosphorylated) form into an active (dephosphorylated) form*”. Their research on DCA was prompted by a previous report in 1962 by Lorini and Ciman that showed that diisopropylammonium dichloroacetate increased oxygen consumption in diabetic rats [113].

Another possible mechanism of action of DCA involved in its antitumor activity is the modification of the intracellular pH that paradoxically has been shown to decrease in glioblastoma cells in mice following an intravenous injection of DCA [114]. Here, we underline the paradoxical issue, since DCA produces a decrease in lactic acid production and an increase in the intracellular pH (pHi) would be expected. This does not happen. On the contrary, pHi decreases (at least temporarily). This means that there must be mechanisms other than lactic acid production affecting pHi. Details of these mechanisms are not yet fully known. 

A different study by Anemone et al. [115] also treated mice with DCA, though in this case, they examined the extracellular pH in breast tumor-bearing mice in vivo. They found that there was an increase in the extracellular tumor pH 3 days after treatment. However, after 15 days, there was resistance to DCA treatment, and the extracellular pH and lactic acid levels returned to the original levels. Although the results of these last two studies have not been reconciled to date, it is tempting to suggest that the more acute effects in these two studies were due to decreased acid extrusion through unknown mechanisms.

In support of this suggestion is a study that showed that the decrease in pHi produced by a single dose of DCA is approximately one-third of the decrease in pHi produced by one injection of cariporide [116] in a glioblastoma model. Cariporide is a Na^+^/H^+^ exchanger (NHE1) inhibitor and its inhibition would decrease the extrusion of protons by this antiporter. 

Several other effects of DCA are known. DCA increases the expression of COX2, and the latter increases tumor resistance to DCA. Therefore, a possible integrated treatment could be administering a COX2 inhibitor with DCA [117]. 

Other actions of DCA are the inhibition of lipogenesis and endogenous cholesterol production. DCA also shows anti-HIF-1α activity. DCA suppresses HIF-1α activity and angiogenesis through the inhibition of PDK-II [118].

**Figure 10:**
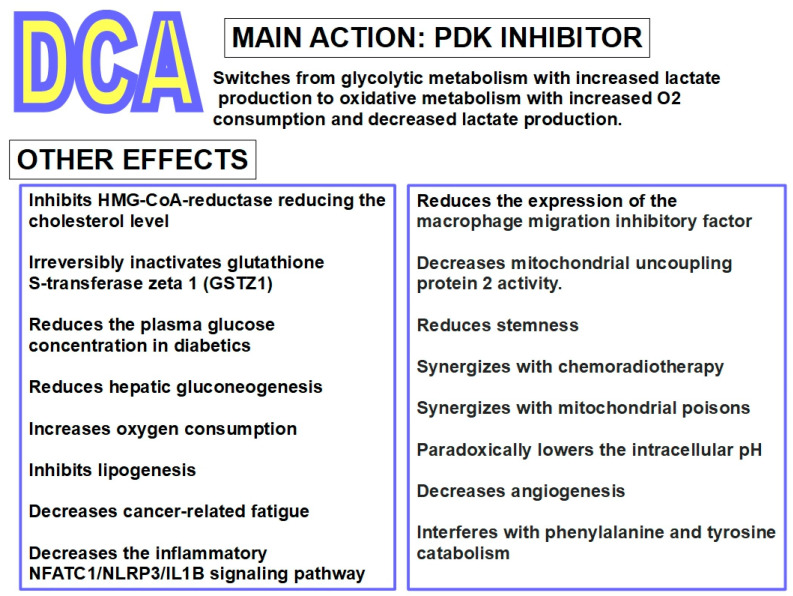
Summary of DCA’s multiple pharmacological effects, many of which stem from PDK inhibition. These include effects on lipid and glucose metabolism, cell stemness, angiogenesis, and pH. The summary is based on references [119,120,121,122,123,124,125,126,127].

Figure 10 summarizes DCA’s pharmacologic effects.

#### Lactylation and DCA

**Figure 11:**
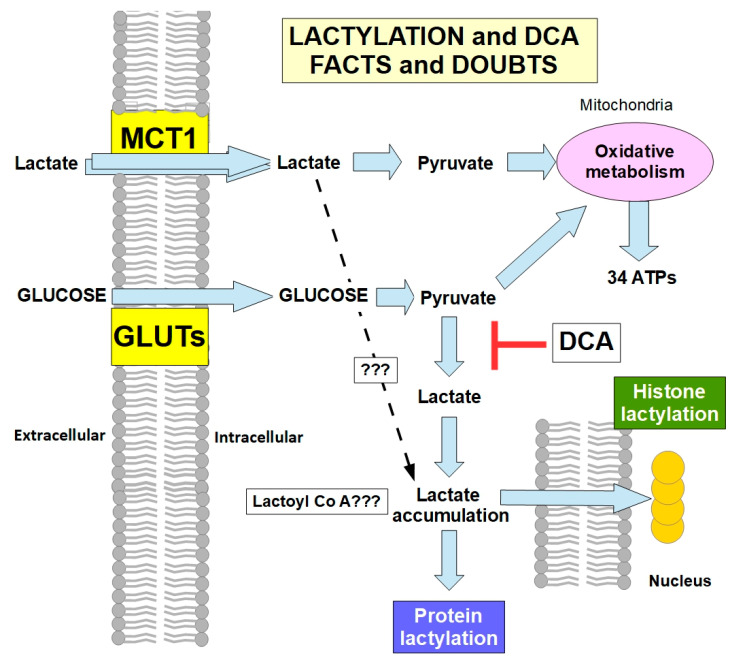
Histone and protein lactylation in cancer and their prevention by DCA are facts supported by evidence [145]. However, there are still certain questions and unknowns. These include the following:

In 2019, Zhang et al. [128] identified histone lysine lactylation (lactyl-amide conjugation) and protein lactylation as effects of lactate accumulation that are able to introduce epigenetic changes and modify the post-translational characteristics of some proteins. Since then, it was found that lactylation participates in many protumoral hallmarks [129,130,131,132,133,134,135,136,137,138,139]. Interestingly, DCA is one of the drugs that can decrease/inhibit lactylation. Wang et al. [140] found that DCA reduced protein lactylation in microglia. Decreased protein and histone lactylation induced by DCA were also found in reparative genes after myocardial infarction [141] and in neuroendocrine type lung and prostate tumors [142], colon cancer cells [143], and lung adenocarcinoma cells [144]. This rapidly accumulating evidence indicates that one of the anticancer mechanisms of DCA is the reduction in lactylation (Figure 11).

(1) Does lactic acid need to be associated with coenzyme A for lactylation [146,147,148]?
(2) Can external lactate that enters the cell through MCT1 activity be integrated into lactylation, or must it be converted into pyruvate? If external lactate (from the lactate shuttle) participates significantly in lactylation, DCA would be ineffective in preventing it. Thus far, there is only evidence of lactylation that originates from lactate metabolically produced by the cell [149]. However, for now, there is no evidence that excludes lactate imported into cells from participating in lactylation.

## 3. Experimental Evidence for DCA Activity in Cancer

Evidence of DCA’s effects on different tumors will be discussed in Section 3.1, Section 3.2, Section 3.3, Section 3.4, Section 3.5, Section 3.6, Section 3.7, Section 3.8, Section 3.9, Section 3.10, Section 3.11, Section 3.12 and Section 3.13.

### 3.1. Breast Cancer

Breast cancer is a malignancy characterized predominantly by glycolytic metabolism [150], which is involved in tumor progression [151] and resistance to therapy [152]. Supporting these observations are the experiments of Godoy et al. [153], who found that 90% of breast cancers had high or very high expression of glucose transporters 1 and 2 (GLUT1 and GLUT2), which means that they have a high glycolytic flux. GLUT5, which is a fructose transporter, was overexpressed in 88% of breast cancers. Furthermore, breast cancer stem cells are also glycolytic [154]. Evidence for the importance of glycolysis in breast cancer was also provided by a study that showed that the inhibition of aerobic glycolysis decreased the AKT/mTOR/HIF-1alpha signaling axis and could restore sensitivity to tamoxifen in resistant cells [155]. Additionally, most breast malignancies have been shown to be glycolysis-addicted [156]. Fluorodeoxyglucose PET scanning confirmed this addiction to glycolytic metabolism [157]. Therefore, since DCA promotes glucose oxidation over glycolysis, it is expected to be inhibitory to these tumors. 

Breast cancer is perhaps the type of cancer in which the effects of DCA have been studied most extensively. Experiments in different models examined the effects of DCA alone and in combination with other treatments. A variety of beneficial effects have been found in studies with DCA alone. For example, Sun et al. [158] found that in an in vivo rat model with mammary adenocarcinoma cell injection, DCA greatly (58%) reduced lung metastasis. Additional experiments showed that the growth of breast cancer cell lines was inhibited by DCA in vitro, while non-cancerous control cells were unaffected. The results with cell lines were similar to those of Gang et al. [159], whereby DCA treatment (1–5 mM) of various breast cancer cell lines markedly decreased cell viability but had little effect on non-cancerous cells. In this study, DCA was also shown to reduce extracellular lactate concentrations, indicating a reversal of the Warburg effect. 

Some other studies used DCA alone to treat breast cancer cells. Harting et al. [160] treated canine mammary carcinoma and mammary gland cell lines with DCA and found that cell proliferation was inhibited, without inducing apoptosis. However, this study (and others) used a high dose of DCA (10 mM) that is not clinically achievable. De Preter et al. [161] also used DCA alone and showed that DCA decreased the pentose phosphate pathway and the glycolytic pathway and reduced proliferation in different breast and cervical cancer cell lines. The effects of DCA alone were also confirmed by Sun et al. [158]. DCA treatment of breast cancer cell lines reversed the glycolytic phenotype and inhibited the proliferation of several breast cancer cell lines. In vivo, that study also showed that DCA inhibited lung metastases in rats. In a similar in vivo study in mice, Blackburn et al. [162] showed that in a murine mammary adenocarcinoma model, DCA treatment reduced tumor growth and numbers, and complete regression of one tumor occurred.

Another approach to using DCA in cancer has been to combine it with other compounds to facilitate either their effects or to facilitate DCA’s therapeutic effect. This approach has been used in several studies. For example, Gang et al. [159] showed that DCA can enhance apoptosis induced by PENAO (phenylarsonous acid) in breast cancer cells, but DCA alone did not cause apoptosis or inhibit growth. Similarly, Wang et al. [163] showed that DCA markedly enhanced doxorubicin-induced breast cancer cell death and had antiproliferative effects in vitro. DCA could inhibit doxorubicin-induced autophagy. DCA combination therapy could also inhibit tumor growth in vivo and prolong mouse survival times. Other compounds that DCA has been successfully combined with include tamoxifen [164], where it sensitized MCF7 breast cancer cells to tamoxifen-induced cell death by downregulating epidermal growth factor expression (EGF); metformin [165], where DCA increased oxidative damage and apoptosis in MCF7 and T47D breast cancer cells with a synergistic effect specific to cancer cells; arsenic trioxide [166], which, when combined with DCA, synergistically inhibited breast cancer cell proliferation and induced apoptosis in a panel of cell lines representing various subtypes of breast cancer; and finally pegylated human arginase [167], which, when combined with DCA, showed synergistic antiproliferative effects on triple negative breast cancer cells.

Robey et al. [168] did not find significant advantages by co-administering bicarbonate with DCA in a MDA-MB231 metastatic breast cancer model in tumor-bearing mice.

The above studies are examples of how DCA may enhance effectiveness of some other compounds. A closely related approach is studies in which another compound improved DCA’s effects on cancer cells. For example, Hong et al. [169] showed that knockdown or inhibition of the S6 kinase 1 (S6K1) enhances DCA-induced cell death in various breast cancer cell lines. It should be noted however that here again, high concentrations, up to 20 mM, of DCA were used. 

The observation that some of these types of effects may vary with different breast cancer subtypes is important. One study [170] compared the effects of different glycolytic metabolism inhibitors and DCA on two breast cancer cell lines. DCA showed higher potency against MCF-7 cell growth relative to MDA-MB-231 cells. Some other metabolic inhibitors of glycolysis also had this trend, though not all of them. These results suggest that this general approach to breast cancer treatment may be very dependent on the cancer subtype and that any future treatments may have to be tailored to the cancer subtype.

A few studies have delved into some more detail about DCA’s mechanisms of action, including some of its downstream effects. Lefort et al. [171] examined different breast cell lines (MDA-MB-231 and MCF-7 cells and a normal control cell line, MCF-10A) that were treated with allopurinol in combination with DCA. Treatments of breast cancer cells with DCA and allopurinol resulted in larger changes in metabolites found in the extracellular medium compared with the changes found in intracellular pools. DCA had no metabolic effects on normal breast cells. Apoptosis was low in the three cell lines. Treatment with low doses of DCA did not lead to high level of apoptosis in MDA-MB-231 cells. Allopurinol decreased the metabolic effects of DCA. DCA caused reduced influx of glucose into MCF7 cells and higher glucose levels in the extracellular medium. This study confirmed that DCA has metabolic effects specific to cancer cells, including reduced glucose uptake. A different and more recent study [172] confirmed some of the suspected modes of action of DCA in two triple negative breast cancer cell lines. DCA decreased the phosphorylation of pyruvate dehydrogenase and lowered lactate production. DCA also increased reactive oxygen species production 15-fold in hypoxic cancer cells but not in aerobic cells. Additionally, DCA made hypoxic tumor cells more sensitive to radiotherapy. A very recent study [173] also demonstrated that DCA treatment can also affect the expression of anti-apoptotic genes and oncogenic miRNAs. These were reduced in MDA-MB231 triple negative breast cancer cells treated with 50 mM DCA. DCA also reduced cell viability in a dose-dependent manner. However, again, these concentrations of DCA are very high. 

### 3.2. Prostate Cancer

Prostate cancer has a peculiar metabolism that changes during its progression [174]. Initially, prostate cancer is not a glycolytic type of tumor, rather it adopts a lipogenic phenotype. 

Two differences in prostate cancer metabolism compared to other tumors are the following:

(1) In prostate cancer cells, oxaloacetic acid is not regenerated during the Krebs cycle but is produced from imported aspartate [175];
(2) Prostate cancer is initially not a glycolytic type of tumor, rather it adopts a lipogenic phenotype until some later critical point during its progression when it becomes glycolytic. This last peculiarity in metabolism contrasts with breast cancer, where glycolysis is the predominant metabolic feature from the very early stages. This also explains the reasons why fluorodeoxyglucose positron emission scans are of little help in the initial stages of prostate cancer [176] and become useful in the advanced stages [177].

Despite the unusual metabolic features, DCA has shown therapeutic potential in prostate cancer. An early observation [178] tested the effect of DCA on human prostate cancer cells, and DCA alone had significant cytotoxic effects and was associated with G1 phase cell cycle arrest and increased apoptosis. The combination of DCA with irradiation sensitized cells to radiation’s cytotoxic effects. A study soon after that one [179] demonstrated that the effects of DCA can vary with the prostate cancer cell type. PC3 and DU145 prostate cancer cell lines were unresponsive to DCA, suggesting that their glycolytic phenotype was due to mitochondrial dysfunction rather than inhibition of PDH. LNCaP prostate cancer cells were more oxidative than the other two cell types and were the only ones to respond to DCA treatment with increased oxygen consumption. Much later, Harting et al. [180] examined six canine prostate adenocarcinoma and transitional cell carcinoma cell lines after their exposure to 10 mM DCA. DCA decreased the proliferation of all but one cell line and led to reduced lactate release. Survivin (an inhibitor of apoptosis) expression was decreased and miR-375 (a microRNA that acts as a tumor suppressor) levels were increased. 

Similar to studies on breast cancer cells, several studies have also examined DCA’s effects on prostate cancer cells in combination with other chemotherapy agents. Zeng et al. [181] showed that DCA dramatically enhances the antitumor effect of cisplatin on PC3 and DU145 prostate cancer cells. Olszewski et al. [182] found a similar effect on other tumor cell types.

One report [183] examined the effect of DCA on African American cell lines, since African Americans are known to respond poorly to therapy compared with Caucasian American patients. They found that DCA inhibited cell proliferation in both African American and Caucasian prostate cancer cells. DCA increased taxol-induced cell death in Caucasian cells (only) and sensitized African American cells to doxorubicin. (African American cells were more aggressive and metastatic than Caucasian-derived cells.)

A very recent report [184] used a prostate cancer cell line (PC3) and showed that DCA can inhibit the cancer stem cell-like characteristics of the cells and strongly influence the metabolic pathway of the cells, causing a shift from glycolysis to oxidative phosphorylation. Their conclusion was that by inhibiting the Warburg effect, DCA may be useful in preventing future cancer relapses caused by cancer stem cells. 

Overall, it is clear that like observations in breast cancer, there is significant potential for beneficial effects of DCA either alone or in combination with other therapies for prostate cancer. 

### 3.3. Colon Cancer

The glycolytic pathway is activated in colon cancer cells [185] and it has been shown that most (76%), but not all colon cancers, show overexpression of glycolytic genes, indicating the enhancement of glycolytic pathways. Experiments in which SW480 and SW460 (colorectal cancer cells from patients) were stimulated with glucose resulted in a proliferative response, and the inhibitor of glycolysis, 2- deoxyglucose, had the opposite effect [186]. Therefore, there is a large group of colon cancer patients that may benefit from inhibiting glycolysis.

In this regard, Madhok et al. [187] found that while DCA did not reduce growth of non-cancerous cells, it caused significant decreases in cancer cell proliferation, which were associated with apoptosis and G2 phase cell cycle arrest. It was also found that DCA induced autophagy in human colon cancer cells with ROS production, mTOR inhibition, and reduced lactate excretion. These effects were reversed upon the cessation of treatment [188]. Delaney et al. [189] reported that DCA did not increase apoptosis in colon cancer cells, even though it decreased levels of the anti-apoptotic protein Mcl-1, but DCA treatment correlated with a decrease in proliferation. DCA reduced stemness markers in colorectal cancers and, importantly, it was able to induce ferroptosis by sequestering iron in the lysosomes [190].

There are also several reports examining the synergistic effects of DCA on colon cancer. DCA was found to act synergistically with 5-fluorouracil (5-FU) [191] and restored colorectal cancer chemosensitivity in 5-FU- [192] and oxaliplatin-resistant cells [193]. Olszewski et al. [182] conducted a study in which they examined the effects of DCA in combination with platinum compounds on several cell types, including COLO205 (colon cancer epithelial) cells. DCA only very slightly increased the cytotoxicity of carboplatin and satraplatin to these cells in vitro and was more efficacious in other cell types.

Notably, there is also an anecdotal case report of a 57-year-old female with stage IV colon cancer treated with oral DCA with tumor stabilization for a period of nearly 4 years, without serious toxicity [194].

### 3.4. Melanoma

Melanoma is a tumor with high glycolytic activity [195]. Several studies have shown that glycolytic pathways are increased in melanoma. For example, an immunocytochemical analysis of lymph node metastasis of patients with melanoma showed increased expression of glycolytic genes [196]. 

Additionally, the BRAF V600E is a common melanoma-initiating mutation and these tumors have a particularly high expression level of GLUT1 [197]. 

Like studies with breast cancer and prostate cancer, there have been studies attempting to use DCA to treat melanoma or to improve other treatments for melanoma. Franco-Molina et al. [198] tested the effect of DCA on several different tumor cell lines, including breast cancer, prostate cancer, and melanoma, and they found that murine melanoma cancer cells were the most susceptible to DCA treatment in vitro. DCA induced apoptosis, inhibited invasion, and inhibited angiogenesis. In mouse experiments with melanoma allografts in vivo, DCA reduced the volume and weight of tumors.

Similarly, another study showed that blocking lactate generation with DCA or oxamate (a lactate dehydrogenase inhibitor) in metformin-treated melanoma cells decreased cell proliferation and tumor progression in mice. Inhibition of complex I alone did not induce apoptosis, whereas inhibiting complex I and lactate generation caused metabolic catastrophe-induced apoptosis [199]. Three other studies confirmed positive effects of DCA treatment on melanomas and provided novel insights into the mechanisms involved. Pópulo et al. [200] showed that melanomas overexpress PDKs, which make them a candidate for DCA treatment. In their study, DCA produced modifications of melanoma cell metabolism, decreased glucose uptake and lactic acid production, downregulating proliferation. Additionally, Abildgaard et al. [201] tested the effect of DCA on melanoma cells in combination with BRAF^V600E^ inhibition. DCA alone reduced glycolytic activity and intracellular ATP levels and inhibited melanoma cell growth. Co-treatment of BRAF^V600E^-mutant melanoma cells with DCA and the BRAF ^V600E^ inhibitor vemurafenib induced greater reductions in intracellular ATP levels and cellular growth than either compound alone. DCA was also effective in vitro *against cells with* acquired resistance to vemurafenib. These results suggest that DCA potentiates the effects of BRAF^V600E^ inhibition through the cooperative inhibition of energy production. A third study found that DCA increases the antitumor effects of the chemotherapy agent capecitabine in a study using a mouse melanoma allograft model [202].

Finally, there is one case report of a 32-year-old male with advanced BRAF-positive metastatic melanoma treated with only DCA (500 mg three times a day). He achieved stable disease and shrinkage of the tumor mass for over 4 years. Recurrence occurred after 4 years following failure to comply with the medication [203]. This is an isolated anecdotal case report but clearly gives some weight to the suggestion that DCA might have a role in treating malignant melanoma and needs further controlled study.

### 3.5. Glioblastoma

Glioblastoma is a glycolytic tumor. Studies have shown that there is an elevation of glycolysis that is facilitated by hypoxia-inducible factor [204]. Some evidence that supports the critical role of glycolysis was provided by Zhou et al. [205]. They isolated a highly tumorigenic group of cells resistant to chemotherapy that can be considered human glioblastoma stem cells. These cells had a 100-fold increase in tumor-initiating ability, were very dependent on glycolysis, had a preference for hypoxia, and had decreased mitochondrial respiration. The glycolytic dependency was demonstrated by glycolytic inhibition with a derivative of 3-bromopyruvate, which caused a significant decrease in the viability of the cells. Both stem cells and non stem cells were very sensitive to glycolysis inhibition. Other evidence for the importance of glycolysis in this cell type was provided by Sanzey et al. [206], who showed that interference with the expression of glycolytic enzymes in glioblastoma decreased tumor growth in hypoxic and non-hypoxic tumors. Another study demonstrated that high expression of glycolytic genes in patients with glioblastoma correlates with poor survival [207]. Conversely, inhibition of glycolysis with DCA and inhibition of fatty acid oxidation with ranolazine improved survival in mice [208]. DCA’s effects on glioblastoma are summarized in Table 1.

The studies included in Table 1 showed the positive effects of DCA on the inhibition of tumor growth or synergistic effects with other chemotherapeutic compounds. However, there was no uniform response in all the tumors. The lack of a uniform response of gliomas to DCA is probably due to the metabolic heterogeneity of gliomas, as has been shown by Duraj et al. [227] and other authors [228,229]. Glycolytic areas may coexist with oxidative areas. Oxidative areas would not be responsive to DCA. Experiments with different glioma cell lines show different metabolic profiles, which may account for the different efficacies of treatments. The metabolic heterogeneity of tumors in general is probably related to DCA failures in other cancers as well. Results from humans are small in scale and anecdotal, and are not conclusive and can only be thought of supporting the need for further study. 

### 3.6. Hematopoietic Tumors

Most hematological malignancies suffer metabolic alterations similar to those seen in solid tumors, including elevated glucose uptake, elevated glycolysis, and elevated lactate production [230]. Suppressing glycolysis has shown potential benefits in hematological cancers [231,232,233,234]. Herst et al. [235] proposed the extent of glycolysis in myeloblasts in acute myeloblastic leukemia as a marker of the pretreatment prognosis. 

#### 3.6.1. Myeloma 

Myeloma is a glycolytic tumor [236,237] and therefore a potential target for DCA. Sanchez et al. [238] found that DCA inhibited glycolytic metabolism and increased sensitivity to the proteosome inhibitor bortezomib in myeloma. The combined treatment increased survival in mice. A test of several glycolysis inhibitors, including DCA, also induced apoptosis in myeloma cells [239]. The combination of DCA with bortezomib showed higher cytotoxicity than each drug alone [240]. However, the concentrations used in these experiments were above those achievable at the bedside. DCA concentrations were 10 mM or higher, and increased ROS production required 40 mM DCA. 

In a clinical trial with seven myeloma patients in partial remission, one patient showed a complete response and two had a partial response. The patient with the complete response had higher DCA blood concentrations due to lower GSTZ1 activity and developed severe neuropathy [241]. We may assume that toxic concentrations of DCA are necessary for significant therapeutic results. Again, this study was conducted at a small scale and no benefit can be concluded. 

#### 3.6.2. Lymphoma

The combination of metformin and DCA showed synergistic apoptotic cell death in B-cell lymphocytic leukemia with downregulation of the anti-apoptotic Mcl-1 protein [242]. DCA-dependent tumor regression and chemosensitization to cisplatin were found in Dalton’s lymphoma [243]. 

There are a few case reports regarding DCA in lymphoma. Flavin et al. [244] reported one anecdotal case of a 48-year-old male patient with stage 4 non-Hodgkin’s follicular lymphoma (NHL) who was treated for 3 months with conventional chemotherapy, resulting in a complete remission. Almost one year later, tumors returned in the nasopharynx and neck lymph glands. The patient began self-administering DCA at 900 mg daily, with a PET scan showing complete remission four months later, and the patient remained tumor-free after 1 year. Another anecdotal report is the case of a man with documented relapse after state-of-the-art chemotherapy for non-Hodgkin’s lymphoma, and a significant response to DCA was documented with a complete remission that was ongoing after 4 years [245].

#### 3.6.3. Leukemia

DCA decreased the viability of several types of leukemia cells in vitro in one study [246], and in another study, it decreased viability of primary chronic lymphocytic leukemia B-cell lines [247] while up-regulating the tumor suppressor p53. It also exhibited anti-leukemic activity in p53 mutated B-chronic lymphocytic leukemia cells [248]. DCA potentiated the cytotoxicity of arsenic trioxide against acute myeloid leukemia cells [249]. 

### 3.7. Ovarian, Cervical and Uterine Cancer

High-grade serous ovarian cancers are glycolytic, while other types of ovarian tumors may be of the non-glycolytic phenotype. Advanced stages are more glycolysis-dependent [250]. Evidence for a putative role of DCA in treatment was provided by a study that showed that DCA increased apoptosis in ovarian cancer cells [251,252]. DCA derivatives were also screened in ovarian carcinoma cells, and they were shown to be more cytotoxic than cisplatin in vitro [253].

Cervical cancer is also glycolytic and overexpresses genes involved in glucose metabolism. This includes HPV-16 positive cervical cells [254]. In HeLa cells, which originated from a cervical cancer tissue sample, DCA shifted the metabolism from aerobic glycolysis to oxidation. The change in metabolism led to a drop in the mitochondrial membrane potential and an increase in the levels of apoptotic proteins. Combined DCA and cisplatin chemotherapy exhibited significant synergy in the inhibition of the proliferation of HeLa cells [255].

Endometrial cancer is also associated with elevated glycolysis and with the expression of genes associated with glycolysis that have prognostic value in the disease [256]. DCA treatment was used in several experiments with endometrial cancer cells. Apoptosis could be induced in five low to moderately invasive endometrial cancer cell lines treated with DCA. The treatment had no effect on non-cancerous cells. Two highly invasive endometrial adenocarcinoma cell lines were resistant to DCA-induced apoptosis [257]. A later study also showed that DCA could inhibit cell proliferation and induce apoptosis in three endometrial cell lines [258]. 

### 3.8. Lung Cancer

#### 3.8.1. Non-Small Cell Lung Cancer (NSCLC)

Lung cancers are glycolytic in general, with high glycolytic flux and elevated levels of glycolytic enzymes [259]. However, some metabolic differences can be found between the different lung cancer subtypes. For example, PCK2 (phosphoenolpyruvate carboxykinase 2) is overexpressed in lung adenocarcinoma, and GLUT1 expression is increased in squamous cell carcinoma [260]. The common point in both cases is that glycolysis is a potential target that is stimulated [261].

Several studies have examined the effect of DCA on various types of lung cancer cells. Oylumlu et al. [262] found that DCA induced significant apoptosis in A549 lung cancer cells (adenocarcinoma cells) after 48 hours of incubation. It decreased the expression of PDK1 and increased the expression of PDH1. DCA was also found to interrupt the Warburg effect and decrease proliferation. Another study showed that DCA increases the cytotoxicity of radiation treatment in A549 and H1299 lung cancer cells [263]. 

DCA has also been shown to have synergistic effects on NSCLC cells. It was shown to enhance the apoptotic effect of chemotherapeutics through the downregulation of autophagy in vivo and in vitro in NSCLC cells [264]. Additionally, DCA re-sensitized human lung cancer cells that were resistant to paclitaxel. There was a synergistic effect between the two drugs [265]. Furthermore, DCA showed synergistic effects with EGFR tyrosine kinase inhibitors in vitro on NSCLC cells [266].

DCA was used against two different lines of non-small lung cancer cells, A549 and LNM35. In both lines, DCA reduced cell viability in a dose-dependent manner and reduced the growth of tumor xenografts and angiogenesis. However, it had no effects on migration and invasion. It also showed additive (LNM35)/synergistic (A549) effects with gefitinib and erlotinib [267]. 

A DCA derivative, dichloroacetophenone biphenylsulfone, which is a powerful PDK1 inhibitor, induced apoptosis in NSCLC cells at sub-micromolar concentrations [268].

Regarding lung carcinoid cells, DCA re-sensitized cells resistant to carboplatin [269]. In Lewis lung carcinoma, DCA slightly decreased the proliferation of the primary tumor (30% less) and markedly decreased the number and volume of metastases [270].

**Figure 12:**
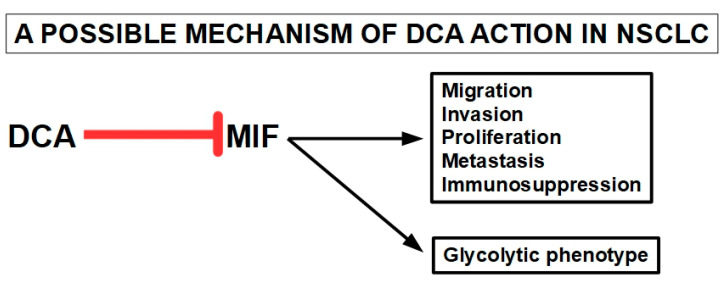
DCA modifies glycolytic metabolism and decreases tumor growth in mice with NSCLC cell xenografts. The DCA-induced downregulation of MIF seems to be an essential antitumor mechanism of this compound in this tumor type.

Feng et al. [271] used integrated metabolomic and transcriptomic studies to elucidate some of the mechanisms of action of DCA in mice with lung cancer xenografts. The comparative metabolomic study between mice with lung cancer treated with and without DCA showed significant differences in the citric acid cycle (Krebs cycle). This indicated that these two groups had different metabolic profiles. A specific genetic analysis showed important differences in the expression of four genes: MIF (macrophage migration inhibitory factor), CLEC3B, FCN3, and EMCN. The DCA-treated group showed considerably smaller tumors, elevated citric acid levels, and a reduction in MIF expression compared to controls. Importantly, Feng et al. [271] confirmed that DCA treatment was able to shift malignant cells away from aerobic glycolysis and increase oxidative phosphorylation. They also postulated an interaction between the lowering of the levels of the cytokine MIF and the modification of the malignant metabolic phenotype. This interaction has been confirmed by other authors [272,273,274,275] (Figure 12).

#### 3.8.2. Small Cell Lung Cancer (SCLC)

SCLC is a highly malignant and metastasizing tumor that represents approximately 15% of lung cancers [276], and it is usually a glycolytic tumor [277]. On the other hand, in this cancer type, the cancer stem cells seem to be predominantly oxidative [278]. Lactate transport in SCLC has been targeted in some experiments [279]. However, we found no publications on DCA treatment of SCLC.

### 3.9. Head and Neck Squamous Cell Carcinoma (HNSCC)

**Figure 13:**
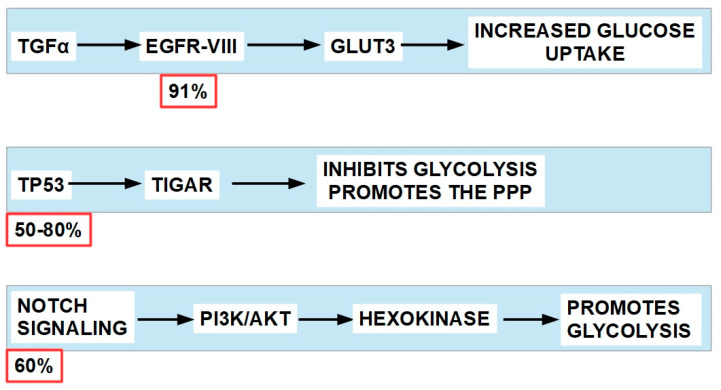
Schematic diagram of activated pathways of the most frequently found mutations in HNSCC and their proglycolytic pathways. Red boxes represent the average percentage of frequency of occurrence of these mutations. EGFR has the highest frequency and is the usual driver gene [284]. Inactivating mutations in the tumor suppressor TP53 deregulate its antiglycolytic effects [285]. Notch signaling recruits hexokinase to the mitochondrial membrane, promoting glycolysis. Inactivating mutations of Notch can also promote glycolysis by depressing oxidative phosphorylation [286,287,288].

Glycolytic tumor-propagating cells drive squamous cell carcinoma, and glycolysis is a main provider of energy in at least some types of the disease [280]. The most frequently mutated/overexpressed driver genes in HNSCC are EGFR, TP53, NOTCH and PI3K [281,282,283]. These genes favor glycolytic metabolism through different pathways (Figure 13).

All the above (Figure 10) confirms that HNSCCs are glycolytic tumors and potential candidates for treatment with DCA. However, a distinction must be made between HPV-negative and HPV-positive oropharyngeal tumors. The former are more glycolysis-dependent and overexpress PDKs, while the HPV-positive tumors mainly utilize mitochondrial respiration and PDK expression is much lower. DCA sensitizes HPV-negative cells to irradiation [289].

Other studies testing the effect of DCA on this type of tumor include the study by Ruggieri et al. [290]. They found that in oral squamous tumors, DCA treatment of three oral squamous cell carcinoma (OSCC) cell lines at pharmacological concentrations resulted in stimulation of oxidative metabolism and caused a remarkably distinct proapoptotic/cytostatic effect on HSC-2 and HSC-3 cells. It also enhanced the production of ROS. Under hypoxic conditions, paclitaxel-resistant OSCC cells were re-sensitized through paclitaxel–DCA synergy. This was not observed in paclitaxel-sensitive cells under normoxia [291]. DCA also achieved the reversal of cisplatin resistance in vitro and in vivo with a concentration of 30 mM [292]. These studies led to a phase II clinical trial, where adding DCA to chemoradiotherapy in HNSCC patients significantly improved the end of treatment response rate but did not modify survival (NCT01386632) [293]. Metformin and DCA acted synergistically, reducing the viability of oral squamous cell carcinoma cells [294].

Overall, HNSCC is clearly another glycolytic tumor type, and DCA has shown beneficial effects and beneficial potential in the treatment of the disease.

### 3.10. Renal Tumors

A study has shown that the Warburg effect is quite pronounced in clear cell renal carcinomas (CCRC) [295], and glycolysis-related gene expression is a potential prognostic marker of CCRC [296].

Most renal cancers (75%) are clear cell renal carcinomas (CCRC). Many CCRCs show a characteristic mutation in VHL (Von Hippel Lindau gene). The VHL protein binds to the transcription factor hypoxia-inducible factor alpha 1 (HFI-α1) and drives its degradation in the proteosome [297]. Mutations of VHL impede HFI-α1 degradation and create a pseudohypoxic condition. Active HFI-α1 translocates to the nucleus, where, in association with HIF-β1, it acts as a transcription factor that activates the expression of more than 300 different genes (hypoxia-inducible genes). Importantly, glucose transporters 1 and 4 and nine of the ten glycolytic enzymes genes are under this HIF regulation. Therefore, CCRCs are glycolytic-dependent tumors that are often dependent on this mechanism [298,299]. 

Several studies have examined effects of DCA on CCRC. Nunez-Xavier et al. [300] found that DCA significantly reduced the viability of CCRC cells but at the same time increased AKT expression. Kinnaird et al. [301] reported that PDK activity was elevated in the tumor compared with normal kidney tissue from the same patient. DCA reduced HIF expression and increased mitochondrial activity with increased ROS levels, increased p53 activity, elevated apoptosis, and decreased proliferation. DCA also reduced viability in a different type of renal cancer cells (G401), Wilms’ tumor, promoting apoptosis and inhibiting migration and invasion. It reduced the expression of glycolysis-related genes [302]. It also reduced the viability of renal cell adenocarcinoma cells (ACHN) in a concentration-dependent manner, inducing G1 phase arrest and apoptosis [303]. In summary, in CCRC, DCA again had beneficial effects on this glycolytic tumor type. 

### 3.11. Pancreatic Cancer

One of the hallmarks of pancreatic ductal adenocarcinoma (PDAC) is aerobic respiration typical of the Warburg effect [304]. Pancreatic tumors also overexpress glucose transporters (GLUTs) like glycolytic tumors. However, PDAC frequently also overexpresses SGLT2, a glucose transporter found in the kidneys. This transporter is inhibited by canagliflozin, a drug in clinical use for the treatment of type 2 diabetes. Inhibition of SGLT2 with canagliflozin has reduced pancreatic cancer growth in mice [305]. 

Several studies have shown potentially beneficial effects of DCA on pancreatic cancer. In one, when different metabolic modifiers were tested against human pancreatic cancer xenografts in mice, the most effective at tumor growth inhibition were phenformin (5 of 12 individuals) and DCA (2 of 6) [306]. DCA and other metabolic modifiers, such as 2-deoxyglucose and phenformin, increased the cytotoxicity of paclitaxel albumin nanoparticles (nab paclitaxel) in pancreatic cancer cells [307]. Another study demonstrated that radioresistant PDAC cells overexpressed PDK and this resistance was reversed by DCA. Importantly, radioresistant cells showed increased glycolysis and high flux towards the pentose phosphate pathway [308]. As expected, in pancreatic cancer cell lines, DCA was shown to increase mitochondrial oxidative metabolism and could decrease glycolysis and non-essential amino acid synthesis [309]. However, according to Tataranni et al. [310], DCA showed cytostatic rather than cytotoxic effects on pancreatic cancer cell lines. DCA also reduced the expression of stemness markers in some cell lines but not in others. Again, to summarize, in these cases of pancreatic cancer, DCA shows beneficial effects, often promoting the effects of other therapeutic treatments. 

### 3.12. Hepatocarcinoma

It was found that DCA enhanced adryamicin cytotoxicity through increased ROS production in hepatocarcinomas [311]. Similar results were obtained with pirarubicin [312]. The co-application of metformin and DCA suppressed human liver cancer cell proliferation, inducing apoptosis through the inhibition of mTORC1 and increased ROS production in vitro and in vivo [313].

### 3.13. Other Tumors

Table 2 summarizes the findings from other tumors not considered above. Generally, the results are similar to those found above with the other cancers, and the effects of DCA include cytotoxicity, glycolysis inhibition, and enhancing the cytotoxicity of other chemo/immunotherapeutic agents. There is variability in the responses; in some cases, DCA did not suppress tumor growth, but in others it did. In some cases, DCA accentuated effect of the therapeutic type of treatments and in others it did not. As noted above, this variability in the effect may have to do with the specific characteristics of the tumors, some being very glycolytic and others being oxidative. Other characteristics of the tumors might also affect the efficacy of treatments, such as multidrug resistance transporters. In these cases, most of the studies have not looked at these other factors and how they affect the effectiveness of DCA treatment. 

## 4. Resistance to DCA

There is little published on this issue. Anemone et al. [115], as commented above, when measuring extracellular pH in vivo, found an initial increase in the extracellular pH of tumors in mice when treated with DCA. After 15 days of treatment, the extracellular pH returned to pretreatment levels. Is there a resistance mechanism to DCA?

On a speculative basis, we should presume that non-glycolytic tumors should not respond to DCA (primary resistance). A similar lack of response would be found in the oxidative cells that are also present in metabolically heterogeneous tumors.

The appearance of resistance after 15 days of treatment with initial response (secondary resistance) should involve a metabolic switch in which the tumors adopt oxidative metabolism and/or a glutaminergic phenotype [326]. To avoid the development of this type of metabolic resistance, DCA should be given simultaneously with other metabolic drugs, such as metformin [327] or 2 deoxyglucose. See below.

**Figure 14:**
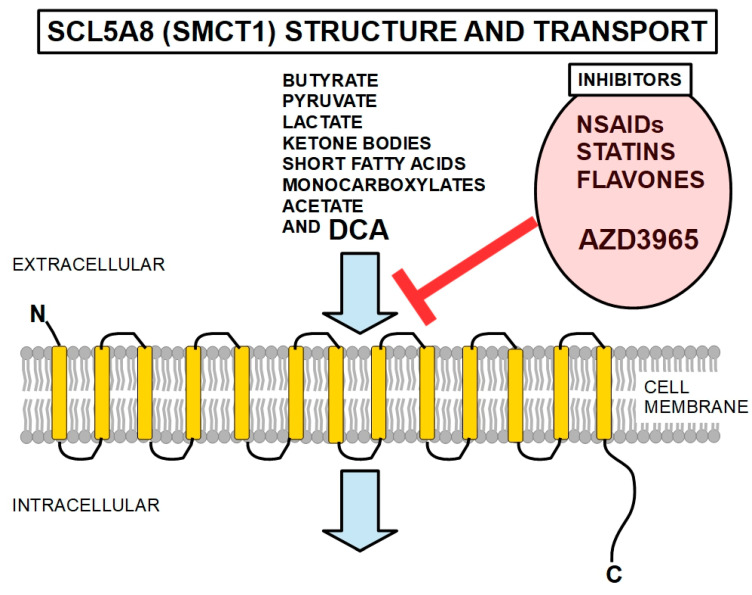
Proposed structure of SMCT1 illustrating the compounds it can transport into the cell on the left side and inhibitors of transport on the right.

SLC5A8 (solute carrier gene family 5a, member 8), also known as SMCT1 or sodium-coupled monocarboxylate transporter 1, is a Na(+)-coupled high-affinity transporter membrane transporter that is considered a tumor suppressor by most authors [328,329]. It facilitates the penetration of DCA into the cell (Figure 11). It mainly transports monocarboxylates, short-chain fatty acids (importantly, butyrate), ketone bodies, pyruvate, acetate, and lactate. SLC5A8 has 610 amino acid residues, and its predicted structure is that of a protein that spans the cell membrane 13 times with a long intracytoplasmic C-terminus and a short extracellular N-terminus [330] (Figure 14).

It has been shown [331] that cancer cells can epigenetically inhibit the expression of SLC5A8, and the authors have proposed the co-administration of a DNA methylation inhibitor to enhance DCA’s effects. This transporter has been shown to be silenced by promoter methylation of the gene in colon cancer [332], papillary thyroid cancers [333], gliomas [334], pancreatic [335], and gastric [336] cancers. Anti-inflammatories in general decrease the transport activity of SCL5A8, and thus they can reduce DCA penetration in the cell.

## 5. DCA and Some Interesting Associations

### 5.1. DCA and Metformin

**Figure 15:**
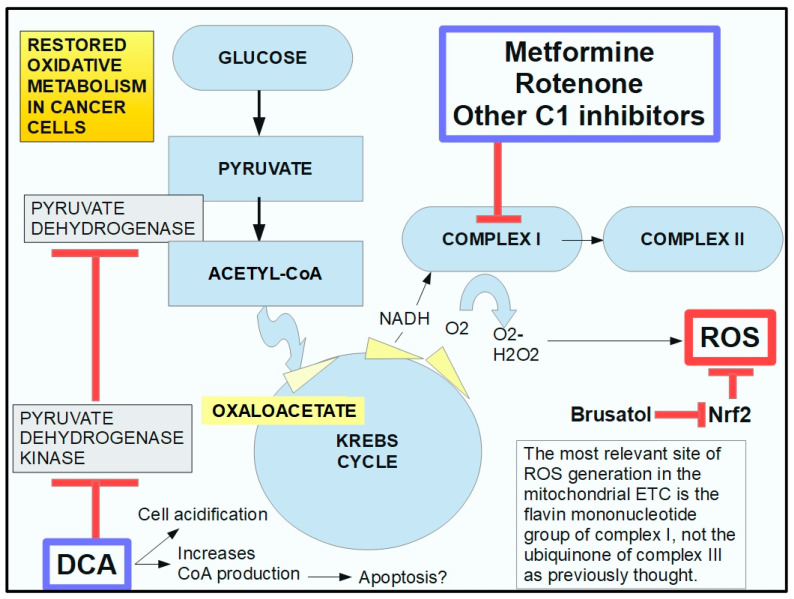
Proposed mechanism by which inhibition of PDK and complex I increases ROS levels and creates oxidative stress. Mitochondria release high levels of superoxide in the presence of electron transport chain (ETC) inhibitors. The figure is based on references [339] and [340] but is controversial. According to Wheaton et al. [341], metformin does not increase the production of reactive oxygen species [342,343], while rotenone does. However, other research shows increased ROS production with metformin [344]. Therefore, the explanation for the synergy between DCA and metformin is still in question. According to other authors, metformin inhibits complex I through ubiquinone inhibition but stimulates ROS production through flavin in complex I [345].

As noted above, DCA in combination with metformin has shown beneficial effects on a number of cell types, including breast cancer cells [165], melanoma cells [199], glioma cells [223,224], lymphoma [242], oral squamous cells [294], and Hela cells [320]. The effect of this combination of compounds was also examined in some detail by Li et al. [337], who noted that the simultaneous use of DCA that decreases lactate production in the cells and metformin that increases lactate production have interesting effects: they synergistically suppress ovarian cancer cell growth and increase apoptosis in vivo and in vitro. Ward et al. [338] searched for an answer to this paradox. They found that in glioblastoma cells, complex I inhibition increases DCA cytotoxicity, whether produced by metformin or other complex I inhibitors like rotenone, through increased oxidative stress. This last finding seems to explain DCA’s cytotoxic activity. It is achieved through an increase in oxidative metabolism that increases oxidative stress, which is enhanced by complex I inhibition. This pathway is shown in Figure 12 that fully illustrates DCA’s mechanism of action, although there are controversies on this issue (Figure 15).

### 5.2. DCA and COX2 Inhibitors

As noted above, Li et al. [117] have demonstrated that inhibition of COX2 enhances the actions of DCA in cervical cancer cells. They also noted that DCA induces COX2 expression, so that DCA could be co-administered with a COX2 inhibitor to achieve better results. On the other hand, COX2 inhibitors may reduce DCA entrance into the cell. This issue is based on indirect evidence of the inhibition of SCL5A8 by NSAIDs.

### 5.3. DCA and Lipoic Acid

Alpha lipoic acid (ALA) is a cofactor of PDH [346] and inhibits PDK [347]. Increased activity of PDH (as produced by inhibition of PDK) should require increased lipoic acid levels. Alpha lipoic acid decreases lactate and pyruvate levels in type II diabetes patients [348], but at the same time, ALA is a strong antioxidant and antiapoptotic compound [349]. This last issue may bring some doubts about the convenience of co-administering ALA with DCA treatment. On the other hand, there are publications showing the antitumor effects of ALA [350] and elevated ROS generation through increased oxidative metabolism [351,352,353]. Feuerecker et al. [354] found that ALA was more effective against proliferation, decreasing lactate production, and inducing apoptosis than DCA. 

We think, even lacking all the necessary experimental evidence, that DCA treatment for cancer could be co-administered with lipoic acid and a COX2 inhibitor like celecoxib.

Further experiments on this hypothesis are suggested.

### 5.4. DCA and 2D-Deoxy Glucose (2DG)

As noted above, 2-deoxyglucose, the inhibitor of glycolysis, has shown beneficial effects in experiments with colon cancer [186] and pancreatic cancer cells [307]. In another case, 2DG markedly increased the anticancer effects of DCA on a Lewis lung carcinoma animal model. DCA alone showed a reduction of 60% of metastasis in this model and a decrease of 90% of the metastasis volume. DCA and 2DG did not affect primary tumor growth, but their co-administration decreased its volume [355].

### 5.5. DCA and Bicarbonate

Robey and Martin [168, [356] found that the chronic co-administration of DCA with sodium bicarbonate to tumor-bearing mice prolonged survival and reduced the tumor volume of re-occurring lesions in the lungs of mice with resected tumors. DCA alone did not achieve any of these results. According to the authors, the reasons for these results are related to a decrease in extracellular acidity. As noted above, Ishiguro et al. [316] used omeprazole, a H^+^, K^+^-ATPase inhibitor that reduces acid secretion, in combination with DCA. They found a synergistic effect on tumors, and this seems to be more than a coincidence. 

### 5.6. DCA and Sulindac

The co-administration of this anti-inflammatory with DCA increased tumor cell killing through increased ROS production [357].

### 5.7. Mitaplatin 

As noted above, a number of studies have shown that DCA in combination with various platinum derivatives can have synergistic effects on a variety of cancer types, including cholangiocarcinoma [325], head and neck cancer [292], lung cancer cells [269], HeLa cells [291], Dalton’s lymphoma [243], retinoblastoma [215], colorectal cancer [192], and some other cell types. The large number of positive results with this combination therapy appears to have led to the development of mitaplatin. Mitaplatin is a fusion compound formed by the association of two molecules of DCA and one of a platinum compound. This allows the simultaneous targeting of DNA and mitochondria. The cytotoxicity of mitaplatin equals that of cisplatin in a variety of cancer cell lines, with the advantage of very low toxicity or no toxicity toward normal cells [358,359]. However, the production of this drug has been discontinued by all the major chemical companies, apparently due to a lack of beneficial effects.

### 5.8. Thiamin

High doses of thiamin (vitamin B1) have effects similar to those of DCA: reduced PDH phosphorylation, reduced lactate production, and increased caspase 3 activity with reduced proliferation of colon cancer cells [360]. This should raise the question: can vitamin B1 replace DCA as a nontoxic PDK inhibitor? Further experimentation with animal models would seem to be warranted.

### 5.9. DCA and Betulinic Acid

Similar to the approach with DCA incorporation into mitaplatin, Saha et al. [361] synthesized a compound whereby DCA was bound to betulinic acid (they called it Bet-CA). They found that this new drug had potent anticancer activity against a wide range of malignant cell lines. It was also tested in vivo in a syngeneic mouse melanoma model and the resulting tumors were much smaller in the treated group than in the control group. Bet-Ca is not being further developed by the pharmaceutical industry.

Betulinic acid (BA) is a triterpenoid that has some anticancer effects. It probably works in a similar manner to metformin in some cases and it paradoxically can also inhibit aerobic glycolysis. Jiao et al. [362] reported that BA inhibited aerobic glycolysis and PDK1 in breast cancer cell lines. It is likely at the crossroad where DCA and BA exert a synergistic mechanism. Additionally, BA was found to be an effective inducer of apoptosis in malignant cells [363,364,365,366,367], while preserving normal cells [368]. It also decreases/inhibits the expression of the protumoral Sp1 transcription factor [369].

### 5.10. DCA and Rapamycin

Chen et al. [370] found that rapamycin, an mTORC1 inhibitor, inactivates PDH, thus limiting its activity in cancer cells. However, DCA co-administration with rapamycin increases cellular susceptibility to mTORC1 inhibitors in vitro and in vivo through rebooting PDH activity. This means that DCA eliminates an undesired protumoral effect of rapamycin.

### 5.11. DCA and Vemurafenib

DCA has been shown to increase the effects of vemurafenib on melanoma cells through increased oxidative respiration and ROS production [200,371].

### 5.12. DCA and Ivermectin

Three patients (one with metastatic breast cancer, a second with femur osteosarcoma, and a third with lung adenocarcinoma) were treated with co-administration of DCA, omeprazole, tamoxifen, and ivermectin. All three patients showed a dramatic improvement in all symptoms when ivermectin was added to the scheme [372]. Ivermectin, among its multiple anticancer effects, causes mitochondrial dysfunction with increased ROS production [373]. This means that it probably acts in a similar manner to metformin.

### 5.13. DCA and TRAIL Liposomes

DCA administered with TNF-related apoptosis-inducing ligand (TRAIL) nanoliposomes synergistically increased apoptosis in lung adenocarcinoma, colorectal, and breast cancer cells. The mechanism involved seems to be increased expression of death receptor 5 (DR5) produced by the metabolic shift triggered by DCA [374].

### 5.14. DCA and 5-Fluorouracil (5-FU)

A novel derivative of 5-FU and DCA in the form of a codrug showed higher cytotoxicity in diverse malignant cells [375]. 

### 5.15. DCA and Chemotherapeutic Drugs in General

DCA has shown synergy, add-on effects, or resistance reversal with almost all classic chemotherapeutic drugs, such as paclitaxel, doxorubicin [376], cisplatin [377], and sorafenib [378].

### 5.16. DCA and Salinomycin

Synergy between DCA and salinomycin has been found in colon cancer cell lines [379]. We believe this synergy is, in part, related to additive cytoplasmic acidification. However, the authors of this publication do not agree with this criterion. They say “*we demonstrate that the synergistic effect of compounds may be related to the inhibitory effect of dichloroacetate on multidrug resistance proteins, and in contrast, it is not related to dichloroacetate-induced reduction of intracellular pH*”.

They also suggested that their experiments replacing DCA with ACE showed that a reduced pHi is not a sufficient factor for potentiating the salinomycin effect.

### 5.17. DCA and Propranolol

The beta blocker propranolol has been identified as a cancer-preventive and antitumoral drug [380,381,382,383]. The mechanisms involved are multiple [384]. The propranolol-induced reduction in norepinephrine levels reduces invasion and causes a limitation of oxidative metabolism. This causes malignant cells to be dependent on the glycolytic pathway. When DCA is co-administered with propranolol, glycolytic escape is blocked and there is a resultant synergistic cytotoxicity [385].

### 5.18. DCA and All-Transretinoic Acid (ATRA)

ATRA induces growth inhibition in many tumors [386,387]. In melanoma, one of multiple mechanisms involved is a reduction in oxidative metabolism with a concomitant major dependence on glycolysis, in a similar fashion as propranolol. When DCA is associated with ATRA, the metabolic stress produced increases cytotoxicity [388]. 

### 5.19. DCA and Radiotherapy

Dong et al. [389] found that DCA radiosensitized esophageal carcinoma cells in vitro and in vivo through increased ROS accumulation. Similar radiosensitizing effects were observed on gliomas and breast cancer [390].

### 5.20. DCA and Omeprazol

Toledo et al. [391] found that the DCA–omeprazol association produced a synergic reduction in the viability of human and canine melanoma cells. Both drugs can induce cytoplasm acidification [392] and probably this may explain their synergy.

### 5.21. DCA and 2-Methoxiestradiol

This association, under hypoxic conditions, inhibited the growth and migration of lung adenocarcinoma cells (A549) [393]. While 2-methoxiestradiol reduces the hypoxia effects by reducing hypoxia-inducible factor 1 alpha levels [394] and inhibits mitochondrial oxidative metabolism [395], DCA introduces metabolic modifications that seem to act similarly to the association with metformin.

### 5.22. DCA and Sirtinol

Sirtinol is a SIRT2 inhibitor and has antitumor effects of its own [396]. DCA showed synergistic antitumor effects with sirtinol on non-small cell lung cancer cells in vitro and in vivo [397]. The mechanism involved is probably the synergistic potentiation of oxidative phosphorylation by co-targeting pyruvate dehydrogenase kinase.

### 5.23. DCA and EGFR Tyrosine Kinase Inhibitors

NSCLC treated with EGFR tyrosine kinase inhibitors develops resistance sooner or later. Studies performed in resistant patients showed increased PDK1 expression. With an NSCLC cell model, it was found that DCA added to the therapeutic effects of EGFR inhibitors [398,399].

## 6. DCA and T-Cells

DCA improved T-cell function in tumors by reducing lactate levels in the microenvironment, thus, allowing T-cell activation. DCA improved the performance of adoptive T-cell therapies when used during the in vitro expansion phase [400]. However, DCA’s effects on immunity are not that clear. It can induce the regulatory T-cell (Tregs) differentiation of human lymphocytes. Effector T-cells depend on aerobic glycolysis while regulatory T-cells do not. Therefore, DCA could deviate lymphocyte maturation towards Tregs by inhibiting glycolysis [401,402].

## 7. Side Effects, Toxicity, and Doses

Initially, DCA was hailed in magazines as almost a miracle “cheap and safe” drug that kills multiple cancers. Studies with animals and cell lines provided promising results, and with administration to rats (75 mg/L in drinking water), tumor growth was reversed without apparent toxicity [71]. This led to DCA being proposed as a therapy for many cancers, including glioblastoma. However, like most drugs, the specificity seems to decline with further use (and further investigation). In several cases, neurological problems have been reported, and these are of course closely related to the dose and method of administration.

Patients who receive doses above 25 mg/kg/day may show a mild sedative effect or drowsiness. The most serious published side effect is reversible peripheral neuropathy [403] that was found in a double-blind placebo-controlled study of 30 patients with mitochondrial myopathy, encephalopathy, lactic acidosis, and stroke-like episodes (MELAS) who were administered 25 mg/kg/day [404]. No renal, cardiac, or bone marrow toxicity have been found, though no benefit for the disease was shown. In another study, Brandsma et al. [405] describe a case of severe encephalopathy due to DCA in a 46-year-old male patient with melanoma treated with 15 mg/kg/day.

The incidence of polyneuropathy in children with mitochondrial disease receiving 12.5 mg/kg twice a day is 10% [406], and in adults, it reaches to 86%.

The mechanism producing neuropathy has not been fully elucidated. It is known that DCA can produce dose-dependent demyelination through the reversible inhibition of myelin-related proteins [407]. Based on some evidence, we speculate that DCA-induced inhibition of glycolysis is the cause, and studies have shown that glycolysis is required by myelinating Schwann cells [408,409,410,411]. In theory, it may be possible to reduce DCA’s neurotoxicity by administering antioxidants [412] because oxidative stress is also one of DCA’s effects. However, this requires experimental confirmation. Importantly, peripheral neuropathy is a reversible phenomenon that improves after a few days of drug wash out. As noted above (Section 1.2.3), glutathione transferase zeta 1 (GSTZ1) is important in the metabolism of DCA and polymorphisms of the GSTZ1 gene in humans may enhance DCA toxicity [413].

Other side effects of DCA may be related to unintentional cell death. DCA treatment affects predominantly cancer cells, but non-cancer cells may also be affected, although in a less significant way [414]. However, at high doses, cellular damage is probably not restricted to cancer cells. There is also a report of DCA induction of liver carcinogenesis and lipoperoxidation in rats and mice [415,416]. Here, the doses of DCA in drinking water were up to 2 g/L, which are much higher than others have used, and oral administration was up to 100 mg/kg doses.

There is a fatal case report of bone marrow and liver toxicity with the artesunate–DCA association in a patient with glioblastoma [417]. In this case, an unknown amount of DCA was given by an alternative practitioner, and so a very high dose may have been administered. There is also experimental evidence of potential liver damage induced by DCA in mice [418,419,420,421]. However, these studies have used up to 2 g/L in drinking water or 300 mg/kg acutely and the high doses may account for some of their results. In some cases, the results also may be due to the co-administration of trichloroacetic acid with DCA.

## 8. DCA Dosage

A total of 10 to 50 mg/kg body weight/day has been found to be a safe dose. However, single nucleotide polymorphisms (SNPs) in the gene for the enzyme *GSTZ1* cause difficulties in establishing a universal dose [422], as noted above. 

## 9. DCA Concentrations in Humans and Animals: A Key Issue

With the unpleasant risk of being repetitive, Table 3 shows the maximum concentrations achieved in humans after an IV injection of DCA [86]. These concentrations are on the level of µg or µM. The reason for this repetition is that the DCA concentration in tumors is the key issue to understanding whether DCA has or may have any therapeutic effect at the bedside.

The concentrations reached in humans are enough to inhibit PDK2, which requires 0.2 mM (see Figure 5) [104]. PDK2 is a widely distributed isoform of the enzyme and the main player in cancer.

Another study showed completely different results. A 25 mg/kg infusion achieved an average level of 129 ± 73 µg/mL on the first day of the infusion, and this increased to 163 ± 84 µg after the fifth infusion [89]. A 50 mg/kg dose achieved a maximum concentration range between 150 and 250 µg/mL [82].

Most of the in vitro studies with DCA were performed at the millimolar or milligram level instead of the micromolar or microgram level. For example, in first study that highlighted DCA’s antitumor activity by Bonnet et al. [71], the lowest DCA concentration was 0.5 mM. The experiments of Sun et al. [158] with breast cancer cells in vitro used DCA at a concentration of 1 to 5 mM. The in vivo experiments carried out in rats had one group that received very high doses (200 mg/kg). According to the estimates of the authors, the plasma concentrations were in the range of 0.5–1 mM with oral administration and 1.5–3.0 mM for intraperitoneal injection. These data are well beyond the maximum concentration peak of 0.15 mM that can be achieved in humans. Furthermore, this is a peak concentration that decreases swiftly after the injection.

The experiments of others also used very high concentrations in vitro. Gang et al. [159] used concentrations between 1 and 5 mM on breast cancer cells. Harting et al. [160] treated canine mammary carcinoma cells with DCA concentrations of 10 mM. At this very high concentration, they found decreased proliferation but no apoptosis. Duan et al. [210] treated C6 glioblastoma cells with DCA concentrations of 5, 10, and 25 mM. They found that apoptosis required an IC50 of 27.0 ± 3.0 mM. At 5 mM, there was no reduction in cell proliferation or apoptosis. Kolesnik et al. [213] showed that under hypoxia, DCA’s cytotoxicity in glioblastoma C6 cells was increased compared with normoxia (IC50 18.2 ± 3.9 mM vs. 51.2 ± 8.1 mM). However, these concentrations are still at levels not achievable at the bedside.

Two other studies used DCA to induce apoptosis in cells. Oylumlu et al. [262] used lung adenocarcinoma A549 cells and found apoptosis at a concentration of 100 mM. Wong et al. [257] studied apoptosis in different endometrial cancer cell lines. Significant apoptosis was only detected with concentrations above 10 mM. Again, both studies used high concentrations of DCA.

A study by Korsakova et al. [219] used somewhat lower concentrations for part of their study on DCA. They found cytotoxicity in glioma cells with a combination of metformin and DCA. The problem is that there were no effects with concentrations of 5 mM DCA and 2.5 mM metformin. The cytotoxic effects were only evident with the higher concentrations of 10 mM DCA and 5 mM metformin. Cytotoxic concentrations could not be reached with non-toxic doses of both compounds. It is of note that even the 5 mM DCA and 2.5 mM metformin concentrations that had no cytotoxic effects were well beyond the levels achievable at the bedside.

Sanchez et al. [238] found that DCA inhibited aerobic glycolysis in multiple myeloma cells at concentrations between 5 and 10 mM, but it required a concentration between 10 and 25 mM to establish increased sensitivity to bortezomib.

Prokhorova et al. [223] demonstrated metformin–DCA synergy in glioma tumors in rats. They administered doses of 1.1 g/Kg DCA and 2.6 g/Kg metformin, which are considered in the therapeutic range, to rats. However, as stated above, this treatment cannot necessarily be extrapolated to humans. The problem is that the DCA concentrations tolerated in rats, mice, and dogs, are very different from those in humans. Therefore, animal experiment results cannot directly be translated to humans. Humans have a lower tolerance to DCA than animals such that the DCA concentrations tolerated by dogs may be toxic in humans.

Other studies that also explored the synergistic effects of chemotherapeutics used concentrations closer to those safely achievable at the bedside. For example, Xie et al. [255] used concentrations of 2, 4, 8, and 16 mM DCA alone. At 2 and 4 mM, very little cytotoxic activity was found. Only at 16 mM did cell viability decrease to 75%, and more significant synergistic effects were only seen with the highest concentrations of DCA.

As to the DCA concentration-dependent cancer effects, it is necessary to further discuss the research by Stockwin et al. [91], who examined non-cancer cells (normal human dermal fibroblasts and human umbilical vein endothelial cells) and some tumor cell lines (transformed MRC5 fibroblasts and a T lymphoblast cell line rho(0) MOLT4 cells). They found that DCA was inactive at concentrations of 17 mM and it only induced apoptosis at concentrations above 25 mM, and this concentration was not cancer-selective, meaning that it was also toxic to normal cells. DCA was cancer-selective when mitochondria were not functional, such as in the rho(0) cells. This explains its synergy with mitochondrial poisons such as metformin and phenformin, and with cisplatin and topotecan that both damage mitochondrial DNA [423,424].

## 10. DCA Derivatives

**Figure 16:**
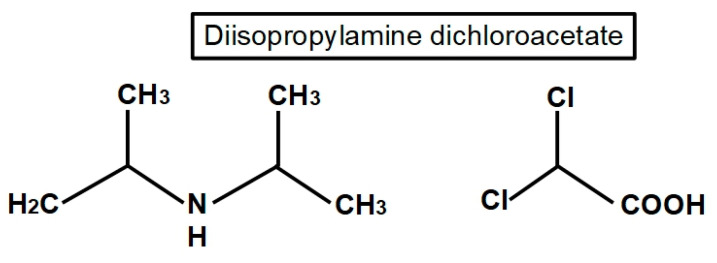
Chemical formula of diisopropyl dichloroacetate.

DCA has been used as the basic part of other chemical structures designed to be more efficient PDK inhibitors. For example, diisopropylamine DCA showed stronger anti-cancer effects than DCA on a xenografted breast cancer model [425] (Figure 16).

In another study, She et al. [426] (Figure 14) synthesized a variety of DCA derivatives combined with arsenicals. Of these compounds, “1f” showed powerful PDK inhibition. The new 1f DCA compound showed high efficacy at a very low concentration (IC50 = 2.0 µM). Furthermore, the authors loaded 1f DCA into nanoparticles that when administered to tumor bearing rats, caused up to 90% tumor shrinkage. Trapella et al. [427] also developed a novel DCA compound they called compound 10, which showed a 30-fold increase in in vitro antitumor activity. This molecule is the result of three DCA moieties bound to a nitrogenated scaffold. DCA compound 10 showed a 30-fold increase in cytotoxicity in vitro [414].

**Figure 17:**
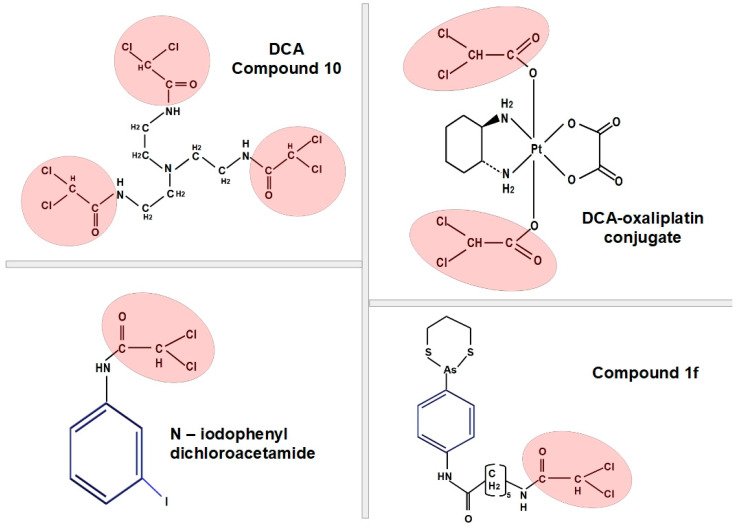
Chemical structures of four different experimental DCA derivatives that have shown increased cytotoxic effects. The DCA moieties are circled.

For a review on DCA derivatives see [428] (Figure 17).

Another DCA derivative is N-iodophenyl dichloroacetamide. This compound has shown high cytotoxicity in tumors in mice with low general toxicity [429]. While a different DCA derivative, DCA-oxaliplatin, overcomes platinum compound resistance and shows increased cytotoxicity. It activates an autophagic response in colon cancer cells [430].

The best known DCA derivative is diisopropylamine dichloroacetate (DADA, Figure 13). DADA is a compound of DCA and diisopropylamine that is sold over the counter for the prevention and treatment of hepatic fibrosis and cirrhosis. It inhibits the activation and proliferation of hepatic stellate cells [431]. It has been proposed as a PDK4 inhibitor for the treatment of the cytokine storm and multiorgan failure [432]. Importantly, DADA has been found to have superior anti-tumor activity compared with DCA in a xenotrasplanted model of breast cancer cells [425]. It requires lower concentrations than DCA to achieve growth inhibition.

## 11. Clinical Cases

DCA has been tested in cancer patients with some promising results. Some of these results have been mentioned above, and these and others are summarized below.

Kahn et al. [433] studied the effects of a DCA intravenous (IV) treatment on patients with different types of advanced cancers. In one patient with colon cancer, there appeared to be an improvement, as indicated by a decline in serum enzyme levels. Another case with metastatic angiosarcoma had mixed responses, some positive, while in a third case, DCA treatment may have stabilized the tumor. The authors concluded that there was some activity in this neuroendocrine pancreatic carcinoma. These studies are interesting, but again only 3 subjects were examined and therefore require further large-scale experimentation. A different study by this group suggested that DCA could stabilize stage 4 colon cancer [194]. Additionally, a third study by the same group showed the stabilization of a patient with metastatic melanoma [203]. The group headed by Khan and working in a DCA clinic reviewed their clinical experience with DCA in patients in a recent publication [434]. Again, these small-scale studies are interesting, but the results must be considered anecdotal evidence

There is also one case in which a patient with non-Hodgkin’s follicular lymphoma who had returning tumors self-administered 900 mg of DCA daily [244]. There was a complete remission in this case, though of course a single such case is not sound or strong evidence of cause and effect. A similar result was shown by Strum et al. [245].

In another study, seven patients with myeloma were treated with DCA, and one patient had full remission and two had remissions [241].

As outlined above [209], the Michelakis group treated 5 patients who had glioblastoma with oral DCA (12.5–25 mg/kg twice daily) for up to 15 months. Three patients showed evidence of tumor regression.

Some other individual cases include one where Ishiguro et al. [435] used DCA together with omeprazol and tamoxifen in a 51-year-old female patient with cholangiocarcinoma in whom no previous standard treatment was effective. They found that the DCA combination therapy blocked further tumor development. Lemmo et al. [436] also described a case of prolonged survival (over a year) after DCA treatment of 500–750 mg/day of leptomeningeal carcinomatosis that originated from NSCLC. As with the studies immediately above, again, these results provide anecdotal evidence only and further clinical trials are needed before any conclusions can be drawn on the efficacy or lack of efficacy of DCA in these kinds of treatments.

### 11.1. Clinical Trials

NCT01111097 [437] was a phase I study to evaluate the safety of DCA in 15 patients with recurrent malignant brain tumors (glioblastoma and brain metastasis of other tumors). Patients were separated into two groups according to genotype, low metabolizers of DCA and quick metabolizers of DCA, with different dose schedules for each group. The starting dose of 8 mg/kg twice daily was increased to 12.5 mg/kg twice daily for the quick metabolizers. No patients suffered toxicities, despite the relatively high dose. The result of the trial showed “clinical and radiological evidence of disease stabilization through the first 4 weeks of DCA administration in all 8 evaluable patients”.

NCT00566410 is a Canadian phase I trial of DCA in 23 patients with solid tumors. No results were published. They established the initial dose at 12.5 mg/kg/day.

NCT05120284 is a multicenter, phase IIA trial of oral DCA in surgical patients with recurrent glioblastoma who have clinically indicated debulking surgery planned. It is ongoing and patients will be randomized to receive DCA (N = 20) or no DCA (N = 20) for one week prior to surgery. Completion is expected in 2025.

#### Clinical Trials with Poor Results

Not all case studies and clinical trials resulted in supportive results. There are in fact several that had poor outcomes. These include several clinical trials. One, NCT01029925 (a USA clinical trials) [438], was a phase I study to evaluate safety of DCA in metastatic breast cancer and lung cancer and a phase II study with seven cases. Only three patients completed 28 days of treatment and only two completed 56 days. Two patients suffered sudden death and another 2 patients had pulmonary embolism. Due to these serious adverse events, the study was terminated prematurely. The dose used in this clinical trial was 6.25 mg/kg twice a day. The authors concluded that that “patients with previously treated advanced NSCLC did not benefit from oral DCA”.

Another clinical trial, NCT01386632 [293], was a study of DCA in combination with cisplatin and radiation for squamous cell carcinoma of the head and neck. A total of 25 patients received placebo and 25 received DCA. Both cohorts concomitantly received radio and chemotherapy. Two-year survival and progression-free survival were similar in both groups. Two patients in the DCA group experienced delirium, but no other serious side events were observed. These results are interesting, as a larger group was studied.

There are other three phase I clinical trials with DCA for cancer treatment, but none have posted results.

## 12. Negative Results with DCA

There are also several studies that have had more negative results with DCA treatment. For example, Hesche et al. [439] evaluated DCA in pediatric tumor cells and a non-malignant cell line and they found that DCA only moderately decreased cancer cell growth. DCA also decreased the cytotoxicity of cisplatin and doxorubicin. It did not activate caspases and decreased caspase activation by cisplatin.

Shahrzad et al. [440] found that DCA had a cytoprotective effect, decreasing apoptosis, in certain cases of colon carcinoma under hypoxic conditions. It also increased the growth of xenografts of SW480 tumors. While there is no clear explanation for this result, it is noted that the effects of DCA may be cell type-specific, and another report has found that DCA can promote the survival of some hypoxic cells [441]. This seems counterintuitive, as DCA inhibits glycolytic metabolism and promotes oxidative flux. It was suggested that in this case, it might support the development of cell clones with more tolerance to hypoxia.

A few other studies found results consistent with these. One study showed that DCA induced tumor radiosensitivity in vitro, but in vivo, DCA decreased radiation-induced growth delays in tumors [442]. In another study of xenotransplanted neuroblastoma in nude mice, progression and proliferation were observed [443]. Anemone et al. [115] followed the effects of DCA in vivo on xerotransplanted mice with breast cancer. They found that initially lactic acid production was decreased, but after 15 days of continuous administration of the drug, lactic acid production and extracellular acidification returned to pretreatment levels. Thus, the effect of DCA can be ephemeral.

There is also an important question about DCA’s effects on the immune system. Many cells of the immune system utilize glycolysis under normal conditions. Can DCA induce an immunosuppressive condition by inhibiting the glycolytic pathway? Dendritic cells are glycolytic, natural killer cells are glycolytic and oxidative, and effector T-cells are highly glycolytic and weakly oxidative, while regulatory T-cells are oxidative [444]. Therefore, in theory, eliminating glycolysis may be detrimental to some of these cell types and may cause decreased antitumor immune activity. Specifically, for example, aerobic glycolysis is necessary for CD4+ T-cell proliferation and differentiation into effector T-cells. DCA can induce apoptosis in these cells or may induce their differentiation towards regulatory T-cells [445]. Therefore, we must conclude that DCA can induce immunosuppression. At the same time, by reducing lactate levels in the extracellular milieu, DCA can counteract lactate immunosuppression improving T-cell functions [446]. Clearly the net benefit or harm of the effects of DCA on the immune system should be further studied.

## 13. Discussion

### 13.1. DCA as a Metabolic Modifier

There is ample evidence showing that DCA is a metabolic modifier that, by inhibiting PDK, restricts glycolysis and enhances mitochondrial oxidative metabolism. This basic mechanism of action has been repeatedly confirmed in different tumors and circumstances, as shown above and in additional references [447,448,449,450,451]. There is evidence that DCA also has other antitumor effects, as summarized in Figure 7. Importantly, all these effects can be achieved with oral administration, with low toxicity and few side effects.

### 13.2. DCA Concentrations

However, the DCA concentration required to reach cytotoxic effects on tumor cells is well beyond those that can be achieved with non-toxic doses in humans. Most of the results of the experiments described above were performed in animal models or cell cultures, where high concentrations can be used. Here, we must insist that these results cannot be extrapolated to humans. Considering the findings of Stockwin et al. [91], the only way to make DCA a useful antineoplastic drug is by simultaneously targeting mitochondria. This could be with topotecan, cisplatin, or by inactivating the electron transfer chain with metformin, phenformin, atovaquone [452,453,454], or some other mitochondrial poison. Furthermore, Robey et al. [168] found that DCA concentrations under 20 mM increased cell viability, and 40 mM or higher concentrations were required to decrease cell viability in vitro. Olszewski et al. [182] found that co-administration of DCA (10 mM) with certain platinum compounds could increase cytotoxicity. However, when the cells were preincubated with DCA alone and subsequently treated with platinum-based drugs, the effect was exactly the opposite. Therefore, when administered with other drugs, the effects of DCA can vary greatly according to the administration schedule.

Table 4 shows examples of the DCA concentrations used in DCA research, whether used alone or associated with other drugs. In many if not most cases, the concentrations used are very high compared to those achievable in vivo in humans (see Table 3).

### 13.3. Effects of Glycolytic Inhibition on Tumor Growth

The question now is can the interruption of the glycolytic metabolism stop tumor growth and induce apoptosis?

To answer this question, we must consider that tumors are formed not only by glycolytic cells but also by oxidative cells. The latter should not be affected by the elimination of the glycolytic pathway. If the tumor is predominantly composed of glycolytic cells, the growth of these cells will be hampered, while the oxidative cells will continue to thrive. However, it must be remembered that even if DCA can stop the proliferation of glycolytic cells, it is unable to induce their apoptosis, unless it is associated with other cytotoxic drugs.

Abundant examples showing that DCA can indeed stop tumor proliferation in different cancer cells in vitro and in vivo have been discussed above. However, these effects are temporary, do not occur in all cancer types, and occur at very high concentrations not achievable in the clinical setting, probably due to some of the following causes:

(a) Cells that survive can switch to another type of metabolism, such as mitochondrial oxidative metabolism;
(b) There may be a persistence of oxidative cells that are not affected by DCA;
(c) DCA is cytostatic rather than cytotoxic, and both require very high concentrations;
(d) The tumor is predominantly oxidative.

The tumor heterogeneity implied above is an important point. As noted, it may account for the wide diversity of results discussed above, where sometimes DCA seems remarkably effective and other times it seems totally ineffective or even harmful.

The in vivo achievable concentration at the tumor site is of course relevant to the clinical effectiveness of DCA. In this regard, Stockwin et al. [91] have shown that DCA requires very high concentrations in the tumor (above 25 mM) to induce apoptosis, concentrations that cannot be reached without intolerable toxicity to normal cells. Therefore, we must conclude that its activity at tolerable doses may sometimes only be cytostatic and may not achieve cytotoxicity. Stockwin et al. [91] also showed that DCA can induce apoptosis in those cells that are unable to switch to mitochondrial oxidative metabolism. Therefore, clearly, the nature and type of the tumor affect the efficacy of DCA. Once again, the tumor type and its heterogeneity will affect DCA efficacy. We suggest that in the rare case studies when DCA administration alone appears to have strong clinically positive effects, this may usually be because it was used in tumors that were highly glycolytic, and possibly with small or negligible components of oxidative tumor cells. There may also be a serendipitous component to some of the results from individual case studies because the studies were anecdotal, without an appropriate sample size and controls. Furthermore, Bianchi et al. [455] reviewed 65 patients in which DCA was used with therapeutic purposes (most of these cases are also included in this review) and concluded that “there is insufficient evidence to affirm that treatment with DCA in cancer patients is effective or is safe”.

### 13.4. DCA Is not a Stand-Alone Drug

A first conclusion is that DCA is not a stand-alone drug because its effects are cytostatic and seem to be temporary, and the tumor cells are not eliminated. Confirming the research by Stockwin et al. [91] is the fact that DCA becomes more efficient when it is co-administered with an inhibitor of oxidative metabolism, such as phenformin or metformin. In this association, the main escape mechanism, that is, switching to mitochondrial oxidative metabolism, is blocked. Another confirmation of the cytostatic rather than cytotoxic effects of DCA is the remarkable synergy between DCA and cytotoxics such as cisplatin. Importantly, Kumar et al. [211] showed the synergistic antitumoral effects of DCA with antiangiogenic drugs such as bevacizumab. We did not find any follow up of this significant finding.

### 13.5. Other Antitumor Effects of DCA

DCA also has other antitumor effects, such as decreasing cell stemness and inhibiting MIF. However, it is not clear if these experimental findings can be utilized without intolerable toxicity at the bedside. Further, we do not know how important these effects are regarding tumor progression. DCA also can decrease mitochondrial uncoupling protein 2 (UCP2) expression, thus increasing ROS production in oxidative cells [456]. Similarly, it is not clear if these effects are exploitable clinically.

We remind the reader of the differences between normal and tumor cells. Under normoxia (normal oxygen conditions), non-cancer cells catabolize glucose to pyruvate. Then, pyruvate is taken up by the mitochondria, where it is further catabolized. As part of this, electrons from the Krebs cycle are taken up by oxygen in the respiratory electron transfer chain. However, tumors are highly hypoxic, and therefore there is very little oxygen for electron uptake, so that this process does not occur or occurs at a reduced level compared with non-tumor cells. The alternative used by tumor cells is anaerobic glycolysis, where pyruvate is not taken up by the mitochondria and instead is converted to lactic acid. The great conductor of this process is hypoxia-inducible factor 1 alpha (HIF-1α). HIF-1α is a transcription factor responsible for the overexpression of more than 300 genes (HIF-responsive genes). PDK overexpression is regulated by HIF-1α [457] and is an adaptation to hypoxic conditions. Evidence for this regulation has been demonstrated. Under hypoxic conditions, when HIF-1α is downregulated in mouse embryos, there is no activation of PDK1. The cell also undergoes apoptosis due to an increase in ROS levels. By re-expressing HIF-1α, apoptosis is avoided and ROS generation is decreased [99].

To summarize these effects, Papandreou et al. [99] state that “The HIF-1 transcription factor drives hypoxic gene expression changes that are thought to be adaptive for cells exposed to a reduced-oxygen environment. For example, HIF-1 induces the expression of glycolytic genes”.

The increased activity of glycolytic genes and enzymes in tumors represents an important resistance factor against apoptosis [458]. DCA’s antitumor effects are significantly increased when used in association with PX-478, a HIF-1α inhibitor [459].

PDH activity represents a key metabolic hub where a “decision” is made as to whether glycolytic metabolism or oxidative metabolism is the next step. PDK is the modulator of this “decision”. In malignant glycolytic cells, the activity of PDH is restrained by the overexpression of PDK. The blocking of pyruvate oxidative mechanism by PDK implies a “functional mitochondrial shutdown”. This is advantageous for malignant cells, because mitochondria produce reactive oxygen species (ROS), which may create oxidative stress. Mitochondrial shutdown avoids this danger. DCA is an inhibitor of PDK, and probably this is its main mechanism of action in cancer.

There is abundant evidence showing that PDK inhibition allows malignant cells to return to oxidative metabolism. In many cases, this return is accompanied by the inhibition of proliferation and even apoptosis. Malignant glycolytic cells are highly dependent on glycolytic metabolism to the point that its reversal to oxidative metabolism causes apoptosis in many, but not all, cases.

As a proof of concept, Li et al. [460] used epigallocatechin 3 gallate (EGCG) (found in green tea leaves) on glycolytic hepatocarcinoma cells, reversing glycolytic metabolism and inducing apoptosis. EGCG acted as an inhibitor of PDK. In the same research, they found that PDK knockdown with siRNAs also induced apoptosis.

**Figure 18:**
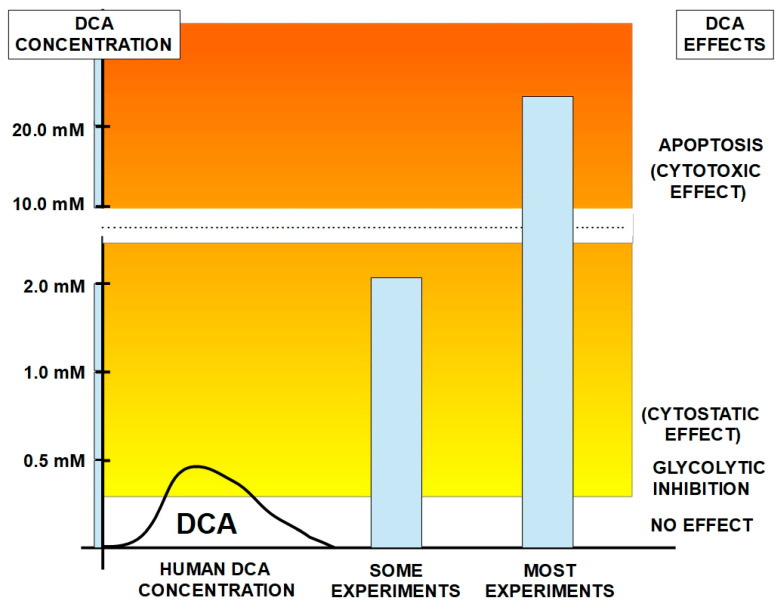
The figure illustrates the maximum tolerable DCA concentration that can be reached safely in humans with the administration of a high but safe dose (left line). The peak concentration can only be maintained for a short time. The few in vitro experiments that showed cytostatic effects were performed with concentrations between 1 and 2 mM (center panel). Most in vitro experiments published in the literature were carried out with concentrations above 10 mM and up to 50 mM (right bar). These may have also shown cytotoxic effects.

Furthermore, DCA concentrations that can be reached in vivo (0.5 mM) seem sufficient to decrease glycolysis and lactate production [461]. However, these concentrations are insufficient for inducing apoptosis (Figure 18).

However, as we said above, DCA alone seems not to fulfill all the requirements needed to be considered a fully chemotherapeutic drug, but when it is used in combination with other specific drugs, the results seem to change.

### 13.6. DCA Associated with Other Pharmaceuticals

There is strong evidence showing that the association of metformin and DCA has significant cytotoxic effects. However, this requires concentrations that are higher than those achievable at the bedside. To this approach, we must add a third compound, a COX2 inhibitor like celecoxib, to decrease COX2 activity induced by DCA. We note that the triple association of DCA, metformin and celecoxib, which has never been experimentally tested in patients, deserves well planned phase II clinical trials.

While even if DCA is far from achieving the cytotoxicity expected from a chemotherapeutic drug, it seems to be an anti-metastatic compound that has properties that can be clinically useful. For example, DCA enhances the cytotoxic activity of the commonly used chemotherapeutic drugs cisplatin, paclitaxel, adryamicin, 5FU, capecitabine, bevacizumab, sorafenib, and doxorubicin, and in many cases, reverses acquired resistance [430,462]. Therefore, DCA should also be considered as a complement to standard chemotherapeutic protocols, especially if chemoresistance has developed.

The intravenous administration of DCA in a once a week or once every two weeks schedule, as performed in many DCA clinics, does not make therapeutic sense. According to its mechanism of action and pharmacodynamic features (short half-life), DCA needs a metronomic scheme (which can use the oral route).

The membrane transporter SLC5A8 seems to play an important role in transporting DCA to the interior of the cell. This transporter usually shows very low expression in malignant cells. However, those tumors that have a normal or increased expression of SLC5A8 show an increased response to DCA treatment [463], possibly due to increased uptake of DCA through this transporter.

Even if it is slightly farfetched, we believe that the glycolytic phenotype in cancer may be due to two different conditions, according to the metabolic response to DCA:

(1) DCA responders—the glycolytic phenotype is due to the upregulation of PDK;
(2) DCA non-responders—the glycolytic phenotype is not dependent on PDK upregulation.

Again, we note that DCA has sometimes fleeting positive effects. In prostate cancer cell lines, Kailavasan et al. [464] showed a major metabolic shift produced by DCA in highly metastatic glycolytic cells, but no changes were found in cell lines with low metastatic potential. The responding cells lacked LDH-B, whereas non-responders expressed both LDH-A and LDH-B. However, the concentrations of DCA used (50 mM) were well above those that can be reached with non-toxic doses in patients. Another example of the elusiveness of the positive effects of DCA is the study by Zwicker et al. [431]. They found that while DCA induced radiosensitization in vitro, it showed no such effects in vivo and in fact showed a slight but significant enhancement of tumor growth in their model in vivo.

The elusiveness of positive effects of DCA in these examples above should also be remembered when proposing DCA for many other diseases, such as diabetes [465,466], pulmonary hypertension [467], ulcerative colitis [468], hyperlipemia [469], prevention of platelet aggregation and thrombosis [470], atherosclerosis [471], endometriosis [472], and amyotrophic lateral sclerosis [473].

Finally, it is important to mention that patients may show variable responses to DCA due to the type of cancer and presence of other diseases. For example, patients with hepatic cirrhosis have a significant decrease in DCA clearance [474] that does not alter the metabolic response to DCA administration [475]. GSTZ1 is downregulated in hepatocellular carcinoma, while it is upregulated in breast cancer [476]. Age also decreases DCA clearance [79].

## 14. Future Perspectives

DCA will never become a stand-alone chemotherapeutic compound. The fundamental reason for this statement is that the drug can only reach micromolar blood concentrations without toxicity and requires millimolar levels to be cytotoxic. It therefore has no chance of becoming a therapeutic alternative. Even when associated with metformin, it still requires clinically unreachable concentrations. However, its synergy with conventional chemotherapeutic drugs opens a window of opportunity that may be exploitable for the benefit of a significant number of patients. Another alternative would be DCA in association with other PDK inhibitors that are under development, such as cryptotanshinone [477,478], oxindole-base-derivatives [479], BX795 [480], amino-triazine derivatives [481], stearic acid [482], and shikonon derivatives [483]. Finally, the toxicity limitation of DCA will probably be improved by novel delivery systems such as antibody–drug conjugates (ADCs). These can release a high dose of a potent cytotoxic agent directly to tumors without affecting normal cells [484]. For example, natural products such as albiziabioside A can be conjugated with DCA, enhancing its effects [485].

ADCs are probably the best way to transform DCA into a real cytotoxic drug because novel and more powerful PDK inhibitors would not be able to circumvent the toxicity involved in PDK inhibition. Some important normal cells depend on functional glycolytic metabolism (e.g., Schwann’s cells and T4 lymphocytes).

## 15. General Conclusions

DCA, when used in toxic doses, decreases tumor growth through the inhibition of PDK and glycolysis and via increased oxidative metabolism with elevated ROS production.

DCA decreases the number and size of metastases in vitro in the laboratory setting.

DCA activity is particularly evident in highly hypoxic tumors.

DCA’s metabolic effects are short lived in vivo.

DCA is not a stand-alone drug and must be used in association with conventional chemotherapeutic compounds or others such as metformin, omeprazol, and bicarbonate. These treatment profiles have not yet been defined.

DCA has synergic effects with most standard chemotherapeutic drugs.

DCA does not induce apoptosis, thus the need for its association with other chemotherapeutics.

DCA should not be used in association with allopurinol, NSAIDs, or flavonoids because they reduce cellular DCA uptake.

To achieve important metabolic changes in cancer cells, DCA must be used for extended periods, at high doses, and on a daily basis. However, there is no evidence of lasting effects.

DCA is not devoid of toxicity, particularly when it is used in adults for a long time and at high doses.

DCA is useful to reverse resistance to chemotherapeutic drugs in highly hypoxic tumors.

Experiments performed in rats or dogs cannot be translated to humans due to important pharmacodynamic differences.

DCA is well absorbed when given orally; therefore, intravenous administration is not necessary.

Cancer cells in which the glutaminergic pathway is upregulated will not be susceptible to DCA treatment.

DCA will probably fail as a stand-alone treatment for tumors with a high proportion of oxidative malignant cells.

DCA, either alone or in association with other compounds, has never been tested in phase II clinical trials and we strongly discourage the use of DCA as a compassionate treatment until well planned and supervised studies are conducted. On the other hand, we believe that DCA deserves more efforts to elucidate its precise role in the treatment of cancer.

Presently, although there is abundant basic experimental evidence for using DCA at high concentrations, this is not sufficient on clinical grounds to support its use due to toxicity at high doses and short-lived effects.

## 16. Main Conclusions

DCA alone reduces glycolytic metabolism in tumors and has a cytostatic effect. However, this is not sufficient to induce apoptosis at achievable non-toxic concentrations in humans.

## Figures and Tables

**Figure 9:**
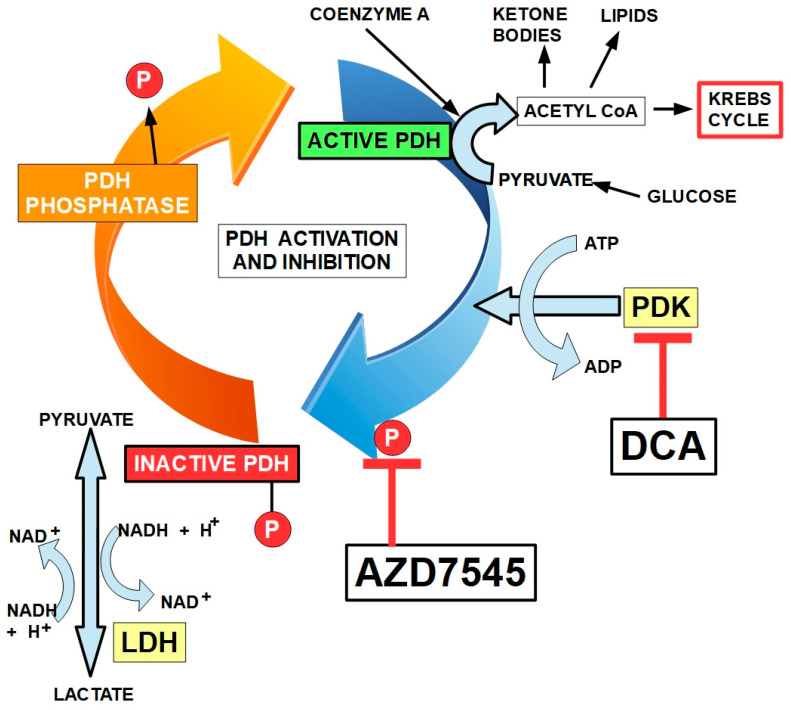
Mechanism of the regulation of PDH (pyruvate dehydrogenase) by PDK (pyruvate dehydrogenase kinase). PDK phosphorylates PDH and inactivates it. PDH phosphatase de-phosphorylates PDH and activates the enzyme. Two different mechanisms of PDK inhibition are shown: DCA and AZD7545 [108]. DCA binding to PDK1 induces local conformational changes that inactivate the kinase activity. Radicicol, another PDK inhibitor, binds to the ATP-binding pocket of PDK3. Inactivated PDH may have up to three phosphorylated sites. Only one is shown in the diagram.

**Table 1:** Glioblastoma—retinoblastoma.

Reference	Findings
Michelakis ED et al., 2010 [209]	The authors tested whether DCA can reverse cancer-specific metabolic and mitochondrial remodeling in glioblastoma. Freshly isolated glioblastomas from 49 patients showed mitochondrial hyperpolarization, which was rapidly reversed by DCA. A separate small experiment treated five patients with glioblastoma with oral DCA for up to 15 months. Three patients showed promising evidence of tumor regression; however, any conclusiuons need repetition with large-scale studies. DCA depolarized mitochondria, increased mitochondrial reactive oxygen species levels, and induced apoptosis in GBM cells, as well as in putative GBM stem cells. DCA therapy also inhibited the hypoxia-inducible factor–1α, promoted p53 activation, and suppressed angiogenesis both in vivo and in vitro.
Duan Y et al., 2013 [210]	DCA inhibited cell proliferation, induced apoptosis, and arrested C6 glioma cells in S phase. In vivo antitumor testing indicated that DCA markedly inhibited the growth of glioma tumors in brain tumor-bearing rats and tumor-bearing nude mice. DCA significantly induced ROS production and decreased the mitochondrial membrane potential in tumor tissues. Antiangiogenic effects were also found.
Kumar K et al., 2013 [211]	DCA synergistically enhanced the results of bevacizumab antiangiogenic treatment in a xenotransplanted mouse model of glioblastoma.
Morfouace M. et al., 2014 [212]	Oct4 is a major regulator of cell pluripotency. DCA increased the amount of Oct4–pyruvate kinase 2 complexes, which inhibited Oct4-dependent gene expression, inducing the differentiation of glioma stem cells.
Kolesnik DL et al., 2014 [213]	Hypoxia enhanced the cytotoxic effects of DCA on glioblastoma cells, inducing necrosis of tumor cells.
Vella et al., 2012 [214]	DCA had anticancer effects on NB (neuroblastoma) tumor cells, which were selectively directed to very malignant NB cells. More differentiated/less malignant NB cells were refractory to DCA treatment.
Sradhanjali S et al., 2017 [215]	DCA reduced retinoblastoma cell line and retinoblastoma explant growth and potentiated carboplatin-mediated inhibition of retinoblastoma cell growth.
Park JM et al., 2013 [216]	C6 glioma was transplanted into rat brains. Magnetic resonance imaging in vivo showed that DCA modified PDH flux much more in gliomas than in normal brains.
Fedorchuk AG et al., 2016 [217]	Experiments in rats with transplanted C6 glioma cells showed that different administration schedules of DCA could cause ambiguous effects: inhibition or stimulation of tumor growth. Prolonged daily administration showed the best antitumor effects. The anticancer efficacy of DCA was significantly increased under hypoxic conditions.
Wicks RT et al., 2014 [218]	Local delivery (wafers) of DCA to experimental brain tumors in rats caused significantly increased survival compared with controls and after oral administration of the drug.
Korsakova et al., 2021 [219]	The authors found synergistic apoptotic effects of metformin and DCA on glioblastomas cells in vitro and in vivo with dose-dependent cytotoxicity. Cytotoxic activity required DCA concentrations above 10 mM and metformin concentrations of 5 mM. There were no effects with concentrations of 5 mM DCA and 2.5 mM metformin.
Shen H et al., 2015 [220]	DCA increased radiosensitivity in orthotopic glioblastoma-bearing mice and of high-grade gliomas, also reviewed in Cook, 2021 [221].
Jiang W et al., 2016 [222]	DCA increased cytotoxic activity of phenformin in glioblastoma stem cells.
Prokorhova IV et al., 2018 [223]	Co-administration of metformin with DCA improved anemia and thrombocytopenia produced by glioma C6 growth.
Kolesnik DL et al., 2019 [224]	It was found that metformin increased the cytotoxic activity of DCA against C6 glioma cells in vitro and in vivo.
Shen H et al., 2021 [225]	DCA associated with metformin and radiotherapy increased apoptosis in pediatric glioma cells in vitro and in vivo. The triple combination of DCA, metformin, and radiation therapy had more potent effects.
Yang Z et al., 2021 [226]	Phosphorylated PDH-A1 mediated tumor necrosis factor-α (TNF-α)-induced glioma cell migration. DCA decreased phosphorylated PDH-A1 levels and reduced glioma cell migration and invasion.

**Table 2:** DCA treatment of other tumors.

References	Organ/Cell	Findings
Ohashi T et al., 2013 [314]	Macrophages	DCA targets macrophages to suppress the activation of the IL-23/IL-17 pathway and prevents arginase 1 (ARG1) expression induced by lactic acid. Lactic acid-pretreated macrophages inhibited CD8+T-cell proliferation, but CD8+T-cell proliferation was restored when macrophages were pretreated with lactic acid and DCA. Although DCA treatment alone did not suppress tumor growth, it increased the antitumor immunotherapeutic activity.
Rooke M et al., 2014 [315]	Sarcoma models in vitro and in vivo	Three types of cells (mouse fibrosarcoma S180 cells, mouse osteosarcoma K7M2 cells and human fibrosarcoma HT1080-luc2 cells) were tested with DCA with and without doxorubicin. DCA alone significantly decreased viability at a concentration of 5 mM. At 0.5 mM, viability only decreased 15%. Importantly, there were no signs of apoptosis; therefore, DCA inhibited proliferation but did not induce apoptosis. DCA had additive effects with low concentrations of doxorubicin.
Ishiguro T et al., 2012 [316]	Fibrosarcoma and colon cancer cells, normal human fibroblasts	The combination of DCA and omeprazol exhibited more potent antitumor activity than DCA alone in tumor cells and did not affect the proliferation of normal cells. Caspase-dependent apoptosis through superoxide production was the suggested mechanism of action.
Sutendra G, et al., 2013 [317]	NSCLC and 2335 mammary cells	In a rat xenotransplanted model, DCA reduced NSCLC and breast cancer tumor vascularity in vivo. DCA also inhibited HIF-1 α in tumor cell lines.
El Sayed SM et al., 2019 [318]	Cancer cells	The authors propose that DCA is an antagonist of acetate, competing for enzymes, and thus its therapeutic action would be caused by acetate deprivation.
Khyzhnyak SV. et al., 2014 [319]	Mouse sarcoma	An examination of sarcoma mitochondria from mice under DCA treatment showed that they had a decrease in lactic acid levels and an increase in PDH activity with a decrease in the electron transport chain activity and an increase in ROS levels.
Choi YW et al. 2014 [320]	HeLa cells	DCA and metformin acted synergistically, enhancing cytotoxicity to cancer cells.
Xuan Y et al., 2014 [321]	Gastric cancer cells	DCA decreased resistance to 5-fluorouracil in highly hypoxic gastric cancer cells.
Badr MM et al., 2014 [322]	Fibrosarcoma	DCA showed immunomodulatory effects through the interleukin 12–interferon γ pathway.
Jin J. et al., 2022 [323]	Osteosarcoma cell lines	DCA mitochondria-targeting micelles induced pyroptosis, increasing the effects of immunotherapy.
Lam SK, et al., 2022 [324]	Mesothelioma	The combination of DCA with niclosamide blocked the growth and proliferation of different mesothelioma cells in vitro and in vivo.
Qin H et al., 2023 [325]	Cholangiocarcinoma	DCA increased sensitivity to cisplatin, changing glycolytic metabolism to oxidative metabolism and increasing ROS levels. Chloroquine further increased this sensitivity.

**Table 3:** DCA dose and plasma concentrations in humans, rats, and dogs, from [86].

Dose in Humans	Concentration Range		Peak Concentration	Half-Life
0	19.9 µg/mL	24.7 µg/mL	0.16 mM	20 min
IV 20 mg/kg	57.3 µg/mL	74.9 µg/mL	0.49 mM	36 min
				
Oral 25 mg/kg			41 µg/mL (0.27 mM) (slow metabolizer)	
Oral 25 mg/kg			30 µg/mL (0.20 mM) (quick metabolizer)	
				
Other sources				
IV 25 mg/kg			130 µg/mL (0.86 mM)	
After 5 infusions			163 µg/mL (1.08 mM)	
				
DOSE IN RATS				
100 mg/Kg	120 µg/mL	164 µg/mL	1.08 mM	4 h
				
DOSE IN DOGS				
100 mg/Kg	447 µg/mL	508 µg/mL	3.36 mM	24 h

Note: The peak concentrations only last for a very short time because the half-life of DCA in plasma is short. Therefore, the average concentrations are far lower than the peak concentrations. Table 3 shows the important differences in DCA’s half-life among species.

**Table 4:** DCA concentrations used in in vitro research.

**DCA ALONE**			
Reference	Authors	**DCA concentration**	
[158]	Sun et al.	5 mM	
[159]	Gang et al.	5 mM	
[160]	Harting et al.	10 mM	
[161]	De Preter et al.	5 mM	
[170]	Xintaropoulou et al.	no response at 1 mM, response above 5 mM	
[178]	Cao et al.	0.5 and 1 mM	
[180]	Harting et al.	10 mM	
[184]	Lai et al.	40 mM	
[187]	Madhok et al.	20 mM	
[188]	Lin et al.	100, 80, and 75 mM	
[189]	Delaney et al.	20 and 50 mM	
[198]	Franco Molina et al.	75 mM	
[209]	Michelakis et al.	0.5 mM	
[210]	Duan et al.	0 to 128 mM, response at 10 and 25 mM	
[214]	Vella et al.	5 and 50 mM	
**DCA CO-ADMINISTERED WITH OTHER DRUGS**			
**Reference**	**Authors**	**DCA concentration**	**Association**
[164]	Woo et al.	20 mM	tamoxifen
[165]	Haugrud et al.	0.5–5 mM	metformin
[166]	Sun et al.	5 mM	arsenite trioxide
[167]	Verma et al.	0.038 mM	arginase
[172]	DE Mey et al.	30, 45, and 60 mM	radiosensitization
[181]	Zeng et al.	5 mM	cisplatin
[182]	Olszewski et al.	10 mM	cisplatin
[191]	Tong et al.	0 to 90 mM	5-FU
[192]	Liang et al.	15 and 20 mM	5-FU
[193]	Liang et al.	15 and 20 mM	oxaliplatin
[201]	Abdilgaard et al.	10 mM	BRAF inhibitor
[211]	Kumar et al.	10 mM	bevacizumab
[213]	Kolesnik et al.	35.8–42.3 mM	hypoxia
[219]	Korsakova et al.	2.5, 5, 10, and 20 mM	metformin
[222]	Jiang et al.	20 mM	phenformin
